# Consensus-Based Sorting of Neuronal Spike Waveforms

**DOI:** 10.1371/journal.pone.0160494

**Published:** 2016-08-18

**Authors:** Julien Fournier, Christian M. Mueller, Mark Shein-Idelson, Mike Hemberger, Gilles Laurent

**Affiliations:** Max Planck Institute for Brain Research, Dept. of Neural Systems, Max-von-Laue-Str. 4, 60438 Frankfurt-am-Main, Germany; McGill University Department of Physiology, CANADA

## Abstract

Optimizing spike-sorting algorithms is difficult because sorted clusters can rarely be checked against independently obtained “ground truth” data. In most spike-sorting algorithms in use today, the optimality of a clustering solution is assessed relative to some assumption on the distribution of the spike shapes associated with a particular single unit (e.g., Gaussianity) and by visual inspection of the clustering solution followed by manual validation. When the spatiotemporal waveforms of spikes from different cells overlap, the decision as to whether two spikes should be assigned to the same source can be quite subjective, if it is not based on reliable quantitative measures. We propose a new approach, whereby spike clusters are identified from the most consensual partition across an ensemble of clustering solutions. Using the variability of the clustering solutions across successive iterations of the same clustering algorithm (template matching based on K-means clusters), we estimate the probability of spikes being clustered together and identify groups of spikes that are not statistically distinguishable from one another. Thus, we identify spikes that are most likely to be clustered together and therefore correspond to consistent spike clusters. This method has the potential advantage that it does not rely on any model of the spike shapes. It also provides estimates of the proportion of misclassified spikes for each of the identified clusters. We tested our algorithm on several datasets for which there exists a ground truth (simultaneous intracellular data), and show that it performs close to the optimum reached by a support vector machine trained on the ground truth. We also show that the estimated rate of misclassification matches the proportion of misclassified spikes measured from the ground truth data.

## Introduction

Recent developments in electrode array fabrication promise to provide neuroscientists access to extracellular signals from hundreds to thousands of neurons simultaneously [1–4]. One related long-standing challenge concerns the computational separation, or sorting, of electrical signals from neuronal populations. Spike sorting consists of clustering waveforms of action potential recorded extracellularly (based on timing, shape and amplitude) so as to assign each cluster to one physical source or neuron [5]. By this means, one can in principle decode a complex multiplexed signal into *n* spike time series, where *n* >> the number of recording channels. This is possible because action potentials produced by each neuron generate relatively stereotypical field potentials that depend on the property of that neuron and on its position relative to each recording site in the array [6,7].

Most spike-sorting algorithms operate in three steps [5,8]: (1) spike waveforms are identified in the voltage trace by threshold detection; (2) a feature set is extracted from the spike-waveform ensemble and used to remap the spikes into a lower dimensional space; (3) spikes are grouped according to their projection along the dimensions of the feature space. As in most clustering problems, spike sorting suffers from the fact that “ground-truth” data are generally absent, making the ranking of potential solutions somewhat arbitrary. Generally, optimizing a clustering solution is assessed relative to some assumption on the distribution of the spike waveforms (e.g., Gaussianity, such as in K-means or Gaussian mixture models) [9,10] and usually relies on heuristic approaches (e.g., re-initialization with new, random initial partitions) to identify a clustering solution that leads to the “best optimum” of an objective function (e.g., maximum likelihood). Most often, the identified clusters also require visual inspection for refinement and validation [11]. Practically, when waveform clusters overlap greatly, the question of whether they should be merged (and thus considered as originating from one single neuron) therefore becomes highly subjective without any reliable quantitative measure of the distance between them [11].

Solutions have already been proposed to estimate the quality of the isolated clusters. However, these measures are either sensitive to the dimensionality of the recording (e.g. isolation distance) or rely on the assumption that spike waveforms are Gaussian distributed.

Here, we propose a new approach to this problem, inspired from consensus clustering methods used in pattern detection and based on evidence accumulation [12,13]. In this approach, the distance between clusters is measured from the reliability of the clustering solution. Consensus clustering based on evidence accumulation takes advantage of the variability in the convergence of the clustering algorithm and identifies the most consensual partition among an ensemble of clustering solutions. While successive iterations of the same clustering algorithm do not generally lead to identical partitions, the variability between different clustering solutions should be lower along the hyper-planes of the waveform ensemble where there is less ambiguity in distinguishing between spike shapes. By looking at the most consensual partition across all clustering solutions, one can identify spikes that are most likely to belong together and therefore correspond to consistent and robust clusters. Because this relies on the probability of spikes clustering together, one can estimate the quality of the final clusters by summing the probabilities of spikes being grouped in one cluster or another. This method has the advantage that it identifies automatically the number of clusters, and does not rely on any statistical model of the spike waveforms.

In the method described here, we identify a large number of clustering solutions using a template-matching approach, in which the templates are defined by K-means clustering of the same spike waveform ensemble with different random seeds. We then compute a distance matrix based on the probability that spikes are localized in the same clusters across all clustering solutions. Using a hierarchical clustering approach over this distance matrix, we then identify groups of spikes that are not statistically distinguishable from one another.

Exploiting preparations that allow simultaneous extra- and intra-cellular recordings, we generated ground-truth datasets and used them to test our algorithm. We show that it approaches the optimal performance reached by a support vector machine trained on the ground truth. We show also that the proportion of misclassified spikes estimated by the consensus clustering approach correlates with the actual proportion of misclassified spikes measured by comparing the sorted spike trains with the ground-truth dataset spike times.

## Results

### Basic principles of consensus-based clustering

**Fig 1:**
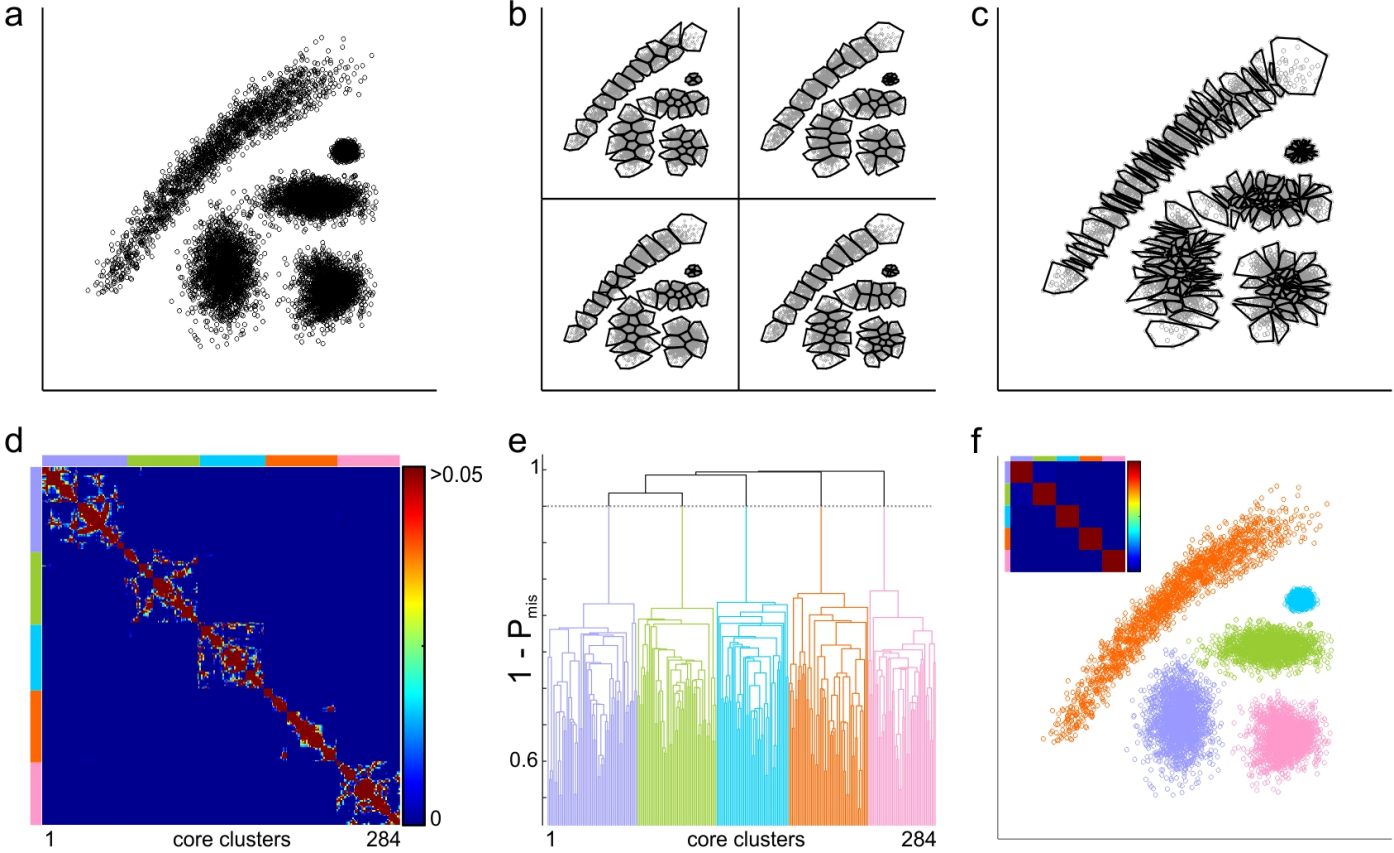
Consensus-based clustering of pseudo-Gaussian clouds. a. Synthetic dataset built from Gaussian distributions. b. Four examples of partitions obtained by K-means clustering with a large number of clusters. The boundaries of each K-means cluster are represented as the convex hull of the clustered points (black lines). c. Core clusters identified from 200 K-means clustering solutions, with a threshold for the core cluster size of 8. d. Pairwise probability of misclassification between core clusters (P_mis_, see Methods). e. Dendrogram obtained by hierarchical clustering with the Single-Link method over the 1—P_mis_ distance matrix. Colors indicate the 5 clusters identified when the cluster tree is cut at 1 –P_th_, with P_th_ = 0.05. f. Final clusters identified by consensus clustering from the dendrogram shown in e. Inset, pairwise probability of misclassification between final clusters.

To illustrate the basic concept of our consensus-based clustering method, we designed a two-dimensional synthetic dataset composed of 5 pseudo-Gaussian clouds, built from multiple distorted 2D Gaussian distributions (Fig 1A). Because K-means clustering is well-suited for hyperspherical clusters, it fails to identify the true boundaries when the expected number of clusters to find is set to 5 (S1A Fig). Using a higher number of clusters in K-means also results in inappropriate clustering since each of the original clusters is split in multiple smaller ones (Fig 1B). However, when the K-means algorithm is run multiple times with a high number of clusters, K-means cluster boundaries tend to lie along those of the original clusters (Fig 1B, S1B Fig). The basic concept of consensus clustering relies on the assumption that points belonging to the same true cluster are more likely to be clustered together across many runs of K-means. Based on the pairwise probability of points being assigned to the same cluster over successive runs of the K-means clustering, we identified a set of core clusters (Fig 1C), corresponding to groups of points which were more consistently clustered together (see Methods). The probability of misclassification between each pair of such core clusters (P_mis_) was then computed from the average number of times a point assigned to one core cluster had been classified with ones assigned to the other core cluster, across all runs of K-means clustering (see Methods). Based on this probability matrix (Fig 1D), we used the Single-Link method to compute a hierarchical cluster tree (Fig 1E) over the distance matrix defined as 1—P_mis_. The corresponding dendrogram was cut at a threshold 1—P_th_, so as to merge core clusters with a probability of misclassification higher than P_th_. Fig 1F shows that the clusters identified at P_th_ = 0.05 successfully match the natural clusters present in the dataset.

The Single-link clustering method consists of a hierarchical sequence of clusters, whereby groups of points are linked together according to the smallest distance observed between any pair of two elements belonging to each of these groups [14]. The main drawback of the single-link method, as classically defined, is the so-called single-link effect: a chain of single points can lead to the merging of distinct clusters. This effect is seen in our example (Fig 1) when the single-link hierarchical clustering method is applied directly to pairwise distances (S1C–S1E Fig). We solve this problem by introducing a threshold on the size of a single link: instead of clustering single data points all at once, we apply Single-Link clustering to ‘core’ clusters, identified by merging together points that are most consistently clustered together. Because we set a threshold on the minimal size of the core clusters (minCsize, see methods), single links with less elements than the size threshold are eliminated. Moreover, the similarity matrix over which the Single-Link clustering is applied takes into account the number of elements in each of the ‘core’ clusters (because it relies on the probability of misclassification); therefore, for equal absolute numbers of misclassified points, smaller ‘core’ clusters will tend to be linked together before being linked to larger ‘core’ clusters. Consequently, the single-link effect is largely reduced and easy to isolate in the dendrogram by adjusting the threshold P_th_.

### Consensus-based clustering of extracellular spikes waveforms

**Fig 2:**
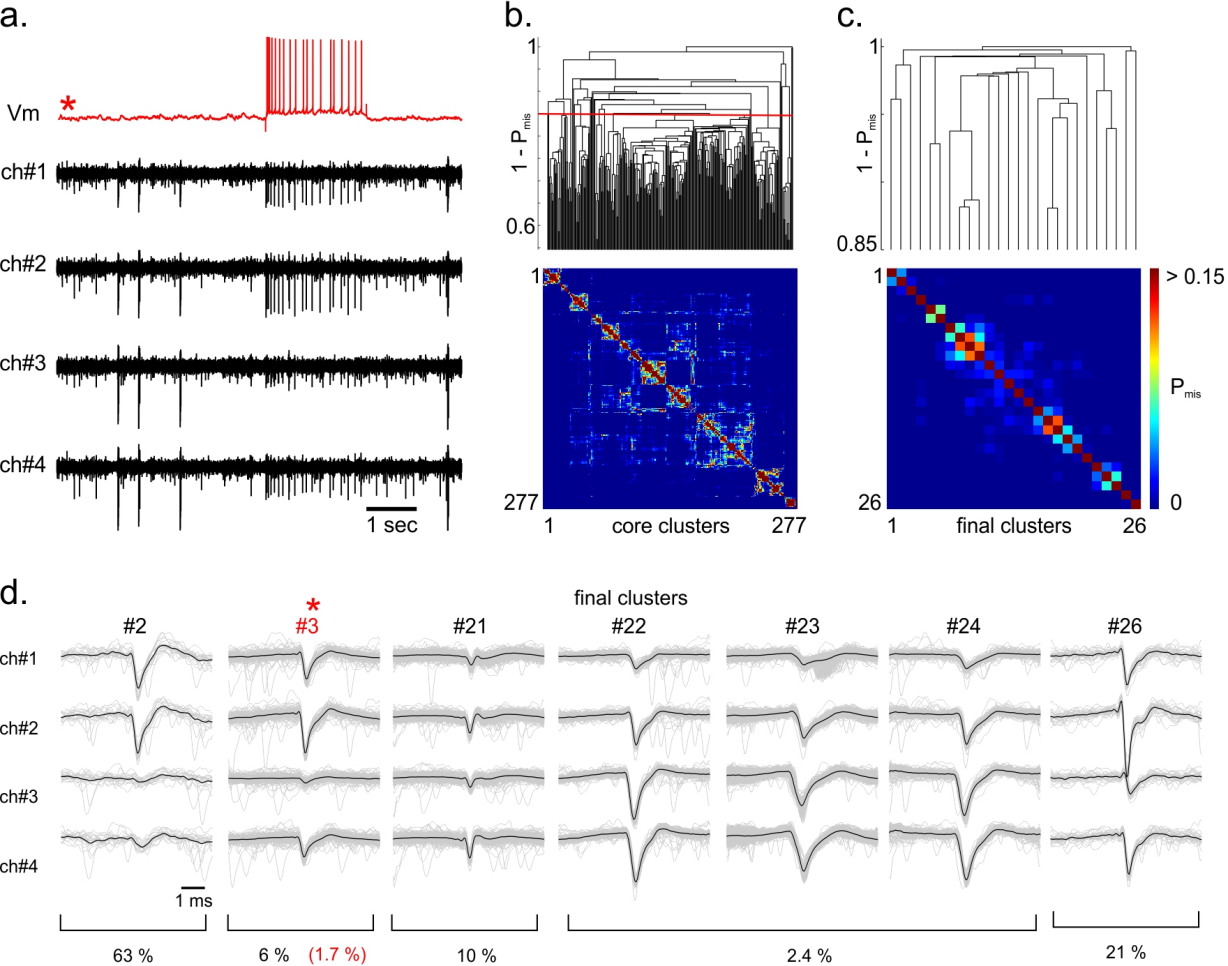
Consensus-based clustering applied to simultaneous intracellular/tetrode recordings. a. Band-passed filtered extracellular voltage signal (black) obtained from a tetrode recording in rat hippocampal CA1. One cell in the vicinity of the tetrode was simultaneously recorded intracellularly (red, [9]). b. Dendrogram (top) obtained by hierarchical clustering with the Single-Link method over the distance matrix (bottom) defined from the pairwise probability of spike misclassification between core clusters. Each leaf of the tree corresponds to a core cluster while the height of each node represents the probability of spike misclassification between the closest core clusters of the linked groups. The final clusters were identified by cutting the cluster tree at 1 –P_th_, with P_th_ = 0.15. c. Dendrogram and distance matrix measured between the final clusters, after consensus clustering. d. Whitened spatiotemporal waveforms of 7 of the final clusters identified by consensus-based clustering. The cluster templates, defined as the average waveforms, are shown in black. Cluster #3 was identified as the single unit matching the intracellularly recorded cell. Spike trains corresponding to cluster #22, #23 and #24 were merged and considered to be the same unit because their cross-correlograms were typical of a bursting cell. The estimated probability of misclassified spikes ($P_{FP}^{total}+P_{FN}^{total}$, see Methods) is shown below each final cluster. After comparing the spike train of cluster #3 to the ground truth spikes, we found a true rate of misclassification of FP_rate_ + FN_rate_ = 1.7%.

The consensus-based clustering method described above was adapted for spike sorting. We used ground-truth datasets (published and new, see Methods) in which extracellular recording had been performed in combination with intracellular recording of one neuron in the vicinity of the extracellular electrode (Fig 2A; [9]). By comparing the spike times of isolated extracellular single units to those of action potentials of the intracellularly recorded neuron, we could assess the accuracy of our spike sorting as the proportion of spike times that were misclassified (see Methods). In short, after detecting the spikes, we performed many runs of a template matching procedure of the spike waveforms based on the templates of K-means clusters obtained with different random seeds. Each iteration consisted of three steps: (1) we used K-means to cluster the spike shapes into a fairly large number of clusters K. This clustering was performed in a reduced feature space, defined by a selected subset of principal components (see Methods). (2) We computed the spatiotemporal averaged templates of all the identified K-means clusters and fitted them to the spike waveforms, allowing for a ±20% variation in the template amplitude (see Eq 2). (3) We assigned each spike to the cluster whose template best explained the spike waveform (in a least-squares sense) and saved cluster labels as well as corresponding χ^2^ errors, before proceeding to the next iteration. For all results presented here, we repeated this template matching procedure 100 times.

We then performed the same consensus clustering procedure as described in the first section, but considering only spikes with an average χ^2^ error below a threshold (χ^2^_th_) defined at 95% of the cumulative distribution of χ^2^ values (S2 Fig). Once the final clusters had been identified by consensus clustering, we fitted the average templates of the ‘final’ clusters to the remaining 5% of the spikes according to Eq (2) and assigned them to the best cluster in the least-squares sense, provided their χ^2^ error was below χ^2^_th_; otherwise, we considered the spike waveform a putative overlap of several spikes and iteratively identified the clusters that best fitted the residual (see Methods).

The advantage of this consensus-based clustering approach is that the diversity of recorded spike shapes represented in the ‘core’ clusters is mapped onto a lower 2-dimensional connection map (dendrogram, Fig 2B) in which each leaf corresponds to a ‘core’ cluster, ordered according to its closest neighbors, while the height of each node represents the distance between them, defined as 1 minus the probability of spike misclassification (1—P_mis_, see Methods) if the connected core clusters had remained separate. Cutting this dendrogram at a certain threshold 1—P_th_ therefore results in a partition where all identified clusters have a pairwise probability of misclassification lower than P_th_.

After visual inspection, the final clusters were validated as well isolated single units if they met 3 criterions: (1) their overall rate of false positive and false negative misclassification ($P_{FP}^{total}$+$P_{FN}^{total}$, see Methods) was less than 20%; (2) their signal-to-noise ratio (SNR, see Methods) was higher than 4; and (3) their rate of refractory period violations (i.e. proportion of inter-spike intervals shorter than 2 ms) was lower than 1%.

In this consensus clustering approach, the threshold for the rate of misclassification (P_th_) needs to be set manually. For all spike sorting presented here, we used P_th_ = 0.15. This fairly high threshold of misclassification rate sometimes led to a sub-clustering of the spike waveforms, requiring manual intervention. Nevertheless, we usually observed better spike-sorting performance with this threshold than with lower values (P_th_ = 0.05 or 0.1 for instance, S3 Fig). Even with P_th_ = 0.15, visual inspection was restricted to cluster pairs with a correlation coefficients greater than 0.7 and a probability of misclassification higher than 0.05. Manual intervention was easy because clusters to be merged were always the closest neighbors in the cluster tree (see for instance clusters #22, #23 and #24 in Fig 2D).

Another parameter of the method is the number (K) of clusters used at each iteration. K should be larger than the true number of single units, to avoid clustering together spikes from different cells. Yet, K should not be too large for this would result in too many small clusters and increase computation time unnecessarily. In practice, we measured the average quality of the fit for all spikes for increasing values of K and chose a value that was high enough to capture most of the variance of the spike waveform ensemble (see Methods, S3 Fig). Spike-sorting performance was stable over a wide range of K values, but higher values of K generally resulted in more clusters, thus requiring more frequent manual intervention (S3 Fig).

### Performances of consensus-based spike sorting on tetrode recordings

We first assessed our method on tetrode recordings performed *in vivo* in rat hippocampal CA1 [6,9]. These recordings were combined with the intracellular recording of one neuron in the vicinity of the extracellular electrode. Fig 2D shows 7 of the clusters identified with our consensus-based spike sorting. Cluster #3 was identified as corresponding to the intracellularly recorded cell. Comparing the spike times of this cluster with the occurrence of an action potential in the intracellular recording, we measured a total rate of misclassified spikes of 1.7% (FP_rate_ = 0%, FN_rate_ = 1.7%). In this recording, another small cluster (cluster #2) had a spike waveform similar to the one corresponding to the intracellularly recorded cell. Our consensus-based clustering correctly identified these few spikes as separate from those corresponding to the intracellularly recorded neuron. Note that clustering the same spike waveforms with a Gaussian mixture model (using an expectation-maximization (EM) algorithm) resulted in incorrect separation of these clusters, whether we used the optimal number of Gaussians as measured from the Bayesian Information Criterion or the number of clusters found by consensus clustering (n = 26, S4 Fig).

We assessed the accuracy of our clustering on 4 other tetrode/intracellular recordings which contained different numbers of “ground-truth” spikes and where the spike templates corresponding to the ground-truth differed in shape and amplitude. The spike sorting was performed using the same parameters as for the recording illustrated in Fig 2.

Spike-sorting performances were compared with the best performances of a support vector machine (SVM) trained on the ground-truth data (see Methods). In this context, the SVM was trained to find the quadratic surface that best separated the ground-truth spikes from the rest of the recording. In other words, SVM searches for features of the spike waveform (the “support vectors”) that are most relevant for distinguishing ground-truth spikes from the rest of the recording. Although SVM cannot be used as a spike-sorting method *per se*, it provides an upper bound on the spike-sorting accuracy that could be achieved with a specific recording. Still, SVM cannot identify overlapping spikes. Therefore, it provides the maximum possible accuracy in the limit of highly overlapping spikes, i.e., when the shape of the ground-truth spike cannot be correctly classified according to the support vectors optimized for the rest of the dataset.

**Fig 3:**
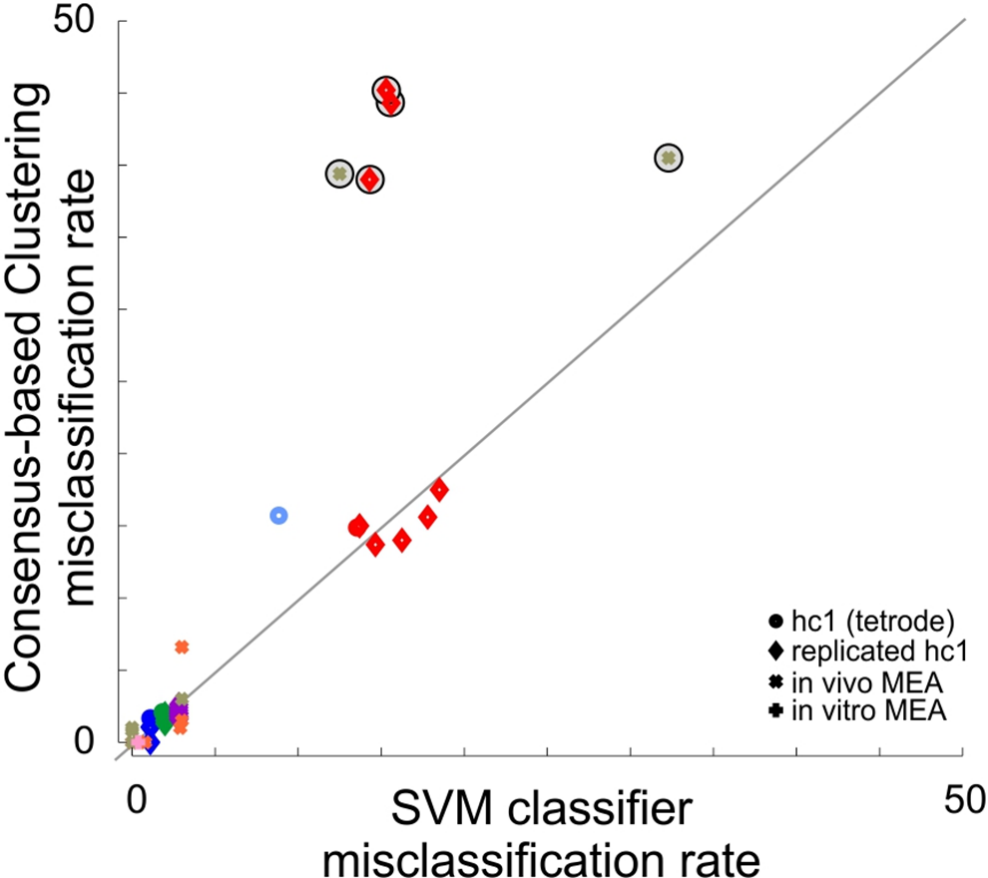
Sorting accuracy. Comparison of the true rate of misclassified spikes (FP_rate_ + FN_rate_) between the consensus-based clustering method and a quadratic support vector machine trained on the ground truth data (SVM, see Methods). Each dot corresponds to the rate of misclassification for the single unit matching the ground truth cell spike times. Symbols and colors correspond to different experiments. A few cells could not be identified correctly and showed much higher rates of misclassification than the optimal performances reached by the SVM classifier. Nevertheless, these clusters (circled symbols) were not validated as well isolated single units because their signal-to-noise ratio (SNR) was too low (SNR < 4) or their estimated rates of misclassified spikes ($P_{FP}^{total}+P_{FN}^{total}$, see Methods) were higher than 20%.

Fig 3 shows that the accuracy of our spike-sorting method on tetrode recordings approached optimal performances reached by the SVM classifier, even for small amplitude single units (those with the highest misclassification rates). After repeating the same spike sorting procedure 10 times, we measured less than 2% standard deviation in the spike-sorting accuracy across different runs of the algorithm. Similar performance was obtained using a single iteration of template matching, with the K-means algorithms set to achieve the global rather than local maximum (S5 Fig). However, our consensus-based procedure had the advantages over such optimized K-means approach that 1) ground truth spikes were less often split in multiple clusters and 2) when required, manual intervention relied on the estimate of the misclassification rates instead of visual inspection alone.

### Performances of consensus-based spike sorting on multi-electrode array recordings

Tetrode recordings can be difficult to sort correctly when neighboring cells generate spike shapes that are very similar (see clusters #2 and #3 in Fig 2D for instance) or when the range of spike amplitudes overlaps the distribution of spikes that cannot be sorted out from the background multi-unit activity (datasets with the highest misclassification rates in Fig 3). Extracellular recordings performed with dense multi-electrode arrays (MEA) can even be more challenging, because they require sorting spikes over 16, 32 or more electrodes at the same time. Although such recordings can provide more spatial information than tetrode recordings, they also result in a considerable increase of dimensionality of the spike waveform ensemble. Moreover, this increase in spatial sampling leads to a higher rate of temporal overlaps between spikes generated at distinct positions of the electrode array.

**Fig 4:**
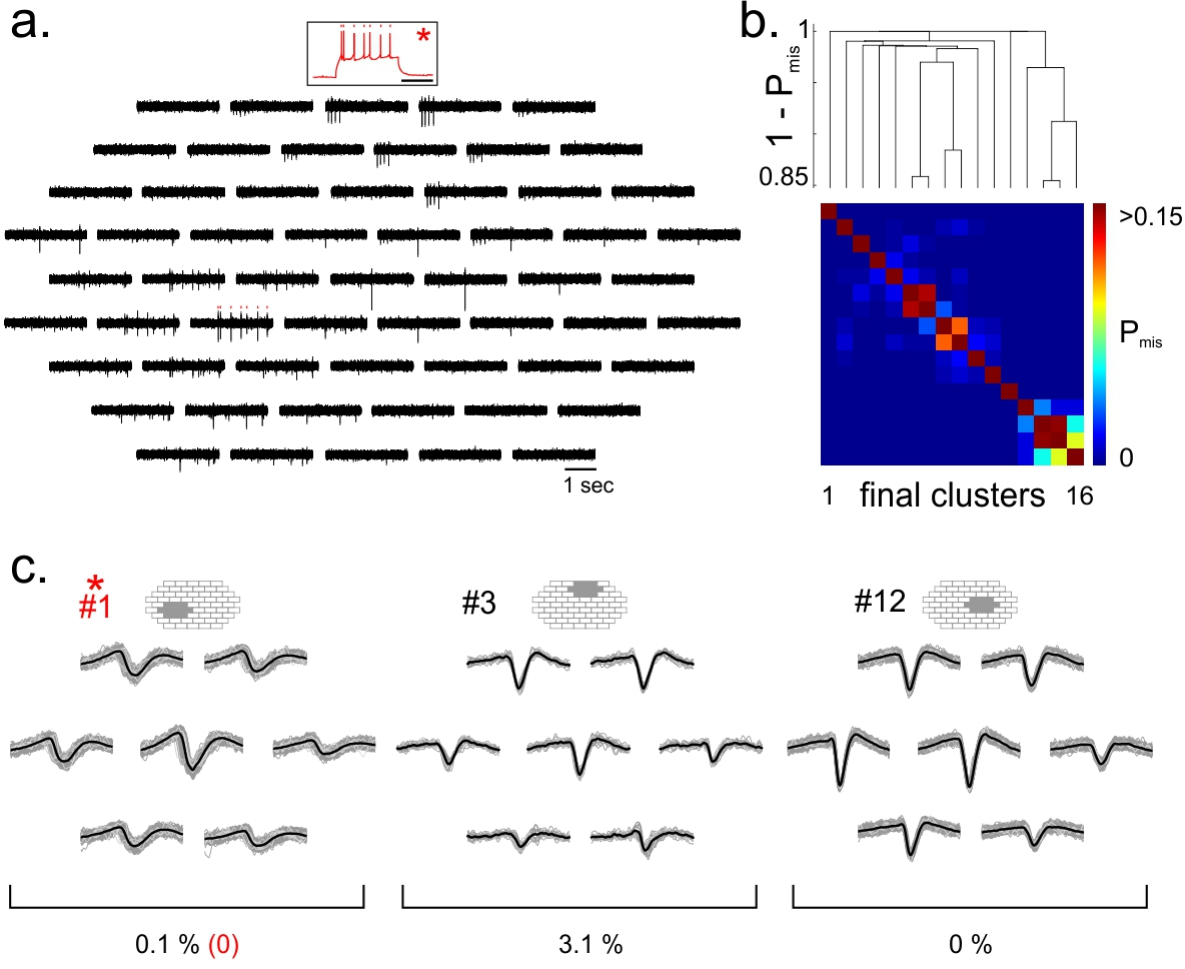
Consensus-based clustering applied to simultaneous intracellular/MEA recordings. a. Band-passed filtered extracellular voltage signal (black) obtained from a multi-electrode array recording (59-electrode MEA, MultiChannel System) performed in vitro in turtle dorsal cortex (Mark Shein-Idelson, Lorenz Pammer, Mike Hemberger and Gilles Laurent, unpublished). One neuron whose spikes could be detected on the MEA was simultaneously recorded intracellularly (*, red). b. Top, Dendrogram obtained by hierarchical clustering with the Single-Link method over the distance matrix defined from the pairwise probability of spike misclassification between final clusters (P_mis_, see Methods). Bottom, Pairwise probabilities of spike misclassification between final clusters. c. Whitened spatiotemporal waveforms of 3 of the final clusters identified by consensus-based clustering. We only show the waveforms over six channels centered on the position of maximal amplitude (shaded area). The cluster templates were defined as the average waveform (black). Cluster #1 was identified as the single unit matching the intracellularly recorded cell. The estimated probability of misclassified spikes ($P_{FP}^{total}+P_{FN}^{total}$, see Methods) is shown for each cluster. All ground truth spikes were correctly classified (FP_rate_ + FN_rate_ = 0%).

We assessed our spike-sorting method on a recording performed with a 59-electrode 2-D array (40 μm pitch, Multi Channel Systems MCS GmbH) on a turtle cortical slab preparation. One of the neurons detected on the array was recorded intracellularly with a whole-cell patch electrode (Fig 4A). When applied to this dataset (using the same parameters as above), our consensus-based spike sorting identified 16 different clusters, among which 9 were validated as single units (Fig 4B and 4C). Importantly, our spike sorting procedure correctly identified the spikes originating from the patched neuron (cluster #1, FP_rate_ = 0%, FN_rate_ = 0%, Fig 4C), despite the variability of the spike shape resulting in a substantial jitter of the spike template.

**Fig 5:**
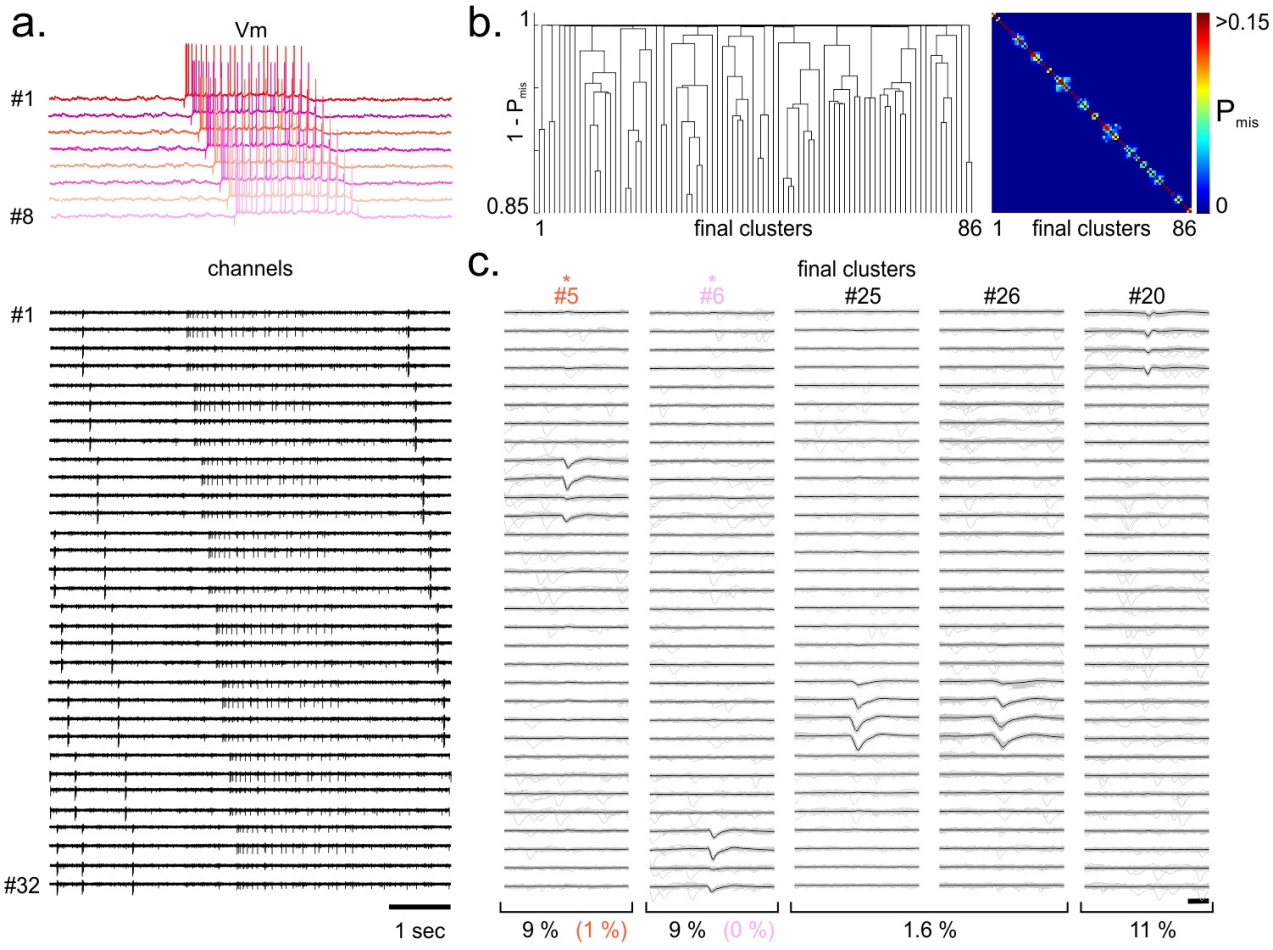
Consensus-based clustering applied to replicated tetrode recordings with ground truth. a. Tetrode recordings obtained from rat hippocampal CA1 with simultaneous intracellular recording from one cell in the vicinity of the electrode were spatially replicated 8 times with a time shift. This resulted in artificial 32-channel recordings with 8 ground-truth clusters. These recordings were processed as if they had been obtained from 32-channel linear electrode array with 20-μm spacing between recording sites. b. Left, Dendrogram obtained by hierarchical clustering with the Single Link method over the distance matrix defined from the pairwise probability of spike misclassification between final clusters (P_mis_, see Methods). Right, Pairwise probability of spike misclassification between final clusters. c. Whitened spatiotemporal waveforms of 5 of the final clusters identified by consensus-based clustering. The cluster templates were defined as the average waveform (black). Cluster #5 and #6 were identified as matching the intracellularly recorded cell #3 and #8 respectively. The estimated probability of misclassified spikes ($P_{FP}^{total}+P_{FN}^{total}$, see Methods) is shown below each cluster. Almost all replicated groundtruth spikes were correctly identified (FP_rate_ + FN_rate_ ≤ 1%).

Because the rate of temporal overlap between spikes from different single units was fairly low, we could not test the resistance of our procedure to this feature. We thus generated fake multi-electrode array recordings by replicating spatially eight times our tetrode datasets and introducing temporal offsets (see Methods). This resulted in virtual 32-electrode array recordings (Fig 5A), with 8 ground truths each. These synthetic ground-truth recordings contained a fairly large number of overlapping spikes; we could thus now assess the accuracy of the spike sorting on overlapping ground-truth spikes.

For the recording illustrated in Fig 5, we identified 86 clusters (Fig 5B and 5C) among which 23 were validated as single units. In this particular example, our spike sorting correctly identified almost all spikes of the 8 ground truths cells (FP_rate_ ≤ 1%, FN_rate_ = 0%) and it took 20 minutes (detection step excluded) to sort the ~37,000 spikes of this 32-channel recording with Matlab 2015a on a computer with 6-cores / 12-threads (Intel Xeon E5-2643 @ 3.4GHz) and 96GB RAM (Ubuntu 12.04.5, Linux kernel 3.13). We applied our spike sorting on 3 other similar recordings and obtained rates of misclassification which were close to optimal performances reached by the SVM classifier for most of the identified single units (Fig 3). Note that some of the ground truth spikes that had small amplitude templates could not be sorted correctly and were clustered together with small amplitude multi-unit activity (Fig 3, circle symbols). Nevertheless, these clusters were not validated as single units because their SNR indicated they were too small to correspond to reliable single unit (SNR < 4). From these datasets, our spike sorting correctly identified all the ground-truth spikes that had occurred at intervals shorter than the censored period (0.6 ms, n = 48). Hence, our algorithm can accurately sort spikes that overlap in time.

**Fig 6:**
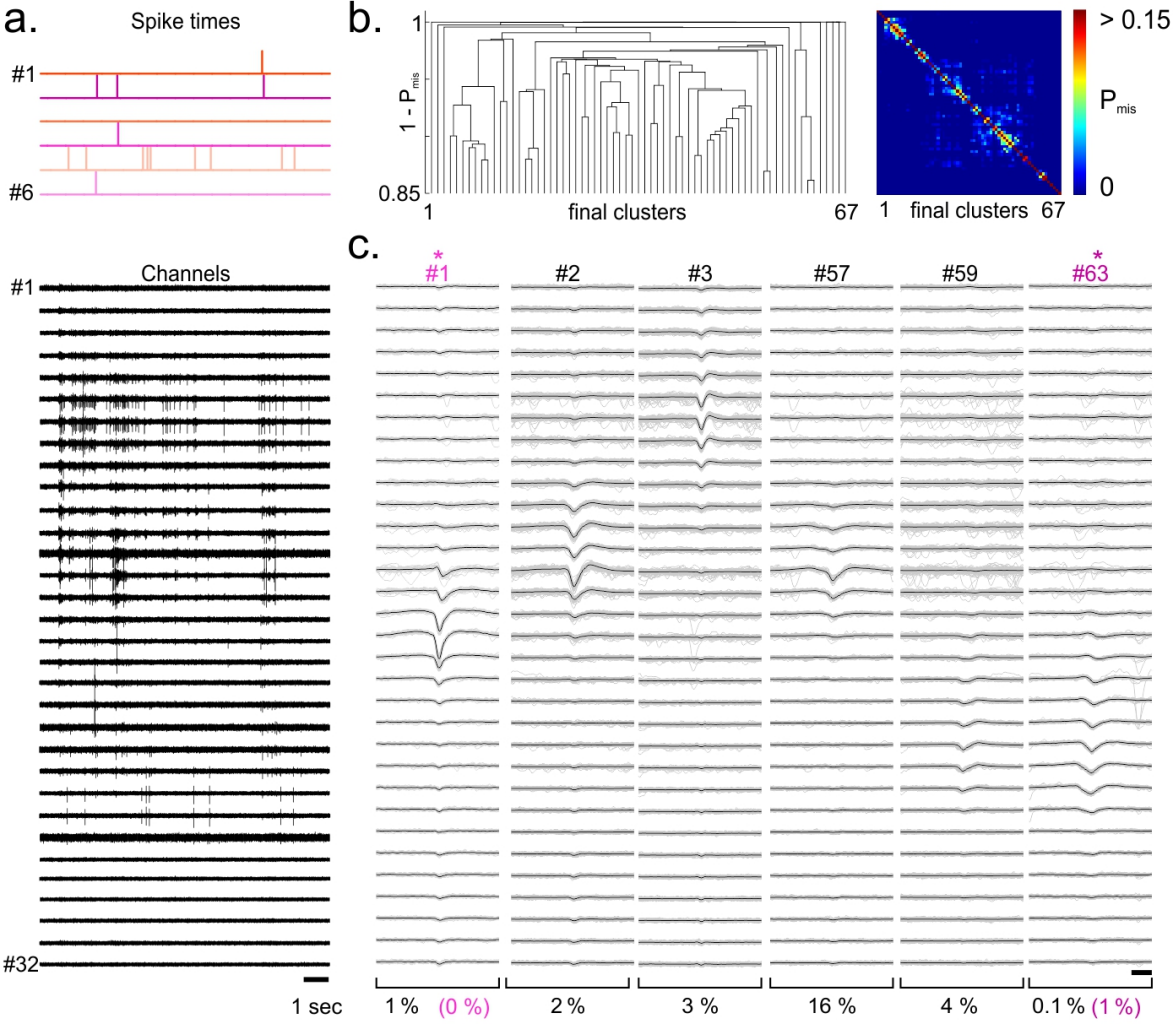
Consensus-based clustering applied to in vivo MEA recordings with artificial ground truth spikes. a. Eight artificial spike trains were added to the raw voltage signal recorded with a 32-channel linear electrode array in the dorsal cortex of the anaesthetized turtle during visual stimulation (see Methods). The artificial spikes waveforms were generated from the cluster template of single units recorded simultaneously but from another electrode array. Artificial spikes were randomly varied in amplitude by ± 10% standard deviation to mimic realistic spike shape variability. b. Left, dendrogram obtained by hierarchical clustering with the Single-Link method over the distance matrix defined from the pairwise probability of spike misclassification between the final clusters (P_mis_, see Methods). Right, Pairwise probability of spike misclassification between final clusters. c. Whitened spatiotemporal waveforms of 6 of the final clusters identified by consensus-based clustering. The cluster templates were defined as the average waveform (black). Cluster #1 and #63 were identified as matching the ground truth spikes of cell #4, and #2 respectively. The estimated probability of misclassified spikes ($P_{FP}^{total}+P_{FN}^{total}$, see Methods) is shown below each cluster, as well as the true rate of misclassification (color).

Finally, we tested our method on *in vivo* recordings performed in the visual cortex of an anaesthetized turtle (Fig 6A, see Methods). For these tests, ground truth was generated by adding to the raw recording, 6 spike trains corresponding to single units recorded at the same time but obtained from another electrode array inserted at a remote cortical site. The artificial spikes were generated from the templates of the corresponding single units, scaled randomly by +/-10% to mimic the variability of single unit recordings. In the example presented in Fig 6, the artificial spikes were correctly identified by our spike sorting procedure and the misclassification rate was close to optimal performance obtained with SVM. Note that two of the artificial single units could not be identified correctly (Fig 3, FP_rate_ + FN_rate_ = 40% and 39%), due to the high similarity of their template with spikes from two real single units present in the recording. The estimated rates of misclassification of these clusters were also indicative of poor isolation ($P_{FP}^{total}+P_{FN}^{total}$ = 29% and 23% respectively).

### Assessment of classification accuracy

**Fig 7:**
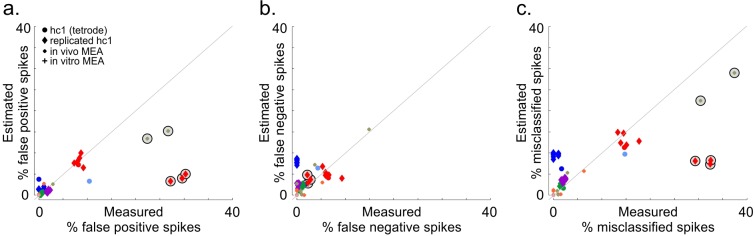
Comparison between estimated and measured rates of misclassified spikes. a. Comparison of estimated rates of false positive spikes ($P_{FP}^{total}$) with actual proportion of false positive error (%FP). Each dot corresponds to the error rate for the single unit matching the ground truth cell spike times. Same symbols and colors as in Fig 3. b. Same as a but for false negative spikes ($P_{FN}^{total}$). c. Same as a but for the total proportion of misclassified spikes ($P_{FP}^{total}+P_{FN}^{total}$). Circled symbols correspond to clusters that were not validated as well isolated single units either because SNR was too low (< 4) or their estimated rates of misclassified spikes was too high ($P_{FP}^{total}+P_{FN}^{total}$ > 20%).

One convenient aspect of the consensus clustering approach is that it relies on the pairwise probability of spike misclassifications between clusters. By measuring the average number of times that spikes from a given cluster are co-localized with spikes of other clusters across all clustering iterations, one can measure the total rate of false positives and false negatives for each identified cluster (see Methods). To assess whether these measures are informative of the true rates of misclassified spikes, we compared the estimated proportion of false positive ($P_{FP}^{total}$), false negative ($P_{FN}^{total}$) and misclassified spikes ($P_{FP}^{total}+P_{FN}^{total}$) to the actual proportion of misclassified spikes (%FP, %FN, %FP + %FN) measured by comparing the sorted spike trains to the ground-truth spike times (Fig 7). The estimated probabilities of false positive, false negative and misclassified spikes were significantly correlated with the actual errors measured from the ground truth (Fig 7A–7C R = 0.86, R = 0.56, R = 0.83 respectively, p << 0.01). We eventually used the total probability of misclassified spikes ($P_{FP}^{total}+P_{FN}^{total}$) to validate the identified final clusters as single units, discarding clusters that had an overall proportion of misclassified spikes higher than 20%.

The Isolation Distance is a common metric used to assess single-unit quality ([9]; see Methods). It relies on an estimate of the covariance matrix of the best Gaussian distribution fitting the spike cluster; this operation is usually carried out in a lower dimensional feature space, into which the full spatiotemporal waveform has been projected. One inconvenient aspect of the Isolation Distance metric, making inter-studies comparisons difficult, is that estimated values depend on the chosen dimensionality. For tetrode recordings, we compared our misclassification-rate estimates with the isolation distance measured on each identified cluster, in a feature space defined by the first 12 principal components (PCs). The total rate of misclassification estimated with the consensus-based clustering was significantly correlated with the Isolation Distance (S6 Fig). The shape of the relationship between these two metrics shows that the estimated misclassification rate provides a more sensitive measure of error rates than isolation distance.

## Discussion

As the techniques to record electrical signals from large numbers of neurons evolve, so do spike-sorting algorithms, providing many potential approaches to the event-detection, feature-extraction and classification problems that such data pose [8]. We developed a new approach to cluster extracellular spike waveforms, which identifies the most consensual partition of spike waveforms among an ensemble of clustering solutions obtained with a standard K-means clustering. The idea of combining the outcomes of different classifiers to obtain robust clusters is not new and has been previously investigated as a valuable clustering strategy for pattern detection and data mining [15]. The same concept has also been used widely in genomic studies to identify expression profiles in gene expression microarrays, by comparing partitions obtained across many subsampled populations of the same dataset [16,17]. To our knowledge, it is the first time that such a method is applied to spike sorting. Originally inspired from consensus clustering methods based on accumulation of evidence [12,13], our method takes advantage of the variability of the K-means clustering (due to sensitivity to initial conditions) to estimate the probability that spikes belong to the same cluster across many clustering solutions. In addition to its relative simplicity, this approach has 2 main advantages over standard spike-clustering algorithms: (1) it does not rely on any statistical model of the spike shapes; (2) it provides an estimate of the probability of misclassified spikes, useful if one wants to assess the quality of the identified clusters.

Classically, spike sorting algorithm using K-means or Gaussian mixture models use heuristic approaches to avoid the problem of convergence to a local, rather than the global optimum. A given algorithm is run several times with different initial partitions, and the solution that converges to the best optimum of an objective function (e.g., maximum likelihood) across all iterations is considered optimal; the others are discarded. Consensus-clustering based on evidence accumulation differs from the above in that it uses all solutions to which the clustering algorithm converged to estimate the probability that one spike is co-localized with another one across all clustering solutions. The decision about which points belong to the same cluster then relies on hierarchical clustering, using the Single-Link method, over the pairwise probability matrix of co-localization in the same clusters across all iterations. In our method, we added an intermediate step to identify a set of ‘core’ clusters corresponding to groups of spikes that were most consistently clustered together before applying the Single-Link clustering. This intermediate stage prevented the so-called single-link effect, for we could apply a threshold on the minimal size of the core clusters to include in the Single-Link clustering. The distance between two core clusters could then be expressed as a rate of spike misclassification (i.e., taking into account the number of spikes in each core cluster) instead of using the absolute number of misclassified spikes. In this sense, our approach is comparable to other algorithms (OPTICS or Density-Linked Clustering) [18,19] that include a density factor in the distance metrics prior to applying hierarchical clustering. It can also be compared to spectral techniques in which clustering relies on a segmentation of a graph of distances between points. Nevertheless, our method differs from these algorithms mainly in that our distance metrics rely on the probability of spikes being assigned to the same clusters across many partitions of the same dataset, rather than on Euclidian distances in some feature space.

We tested the accuracy of our method on different ground-truth datasets and showed that it generally performs close to the optimal levels reached by SVM trained on ground-truth data. Although slightly better performances may be obtained by tuning the parameters of the spike sorting to each of these datasets (such as the cutoff frequencies of the band-pass filter, the detection threshold or the nature of the features used for clustering), we preferred presenting here performances obtained when using the same set of parameters irrespectively of the nature of the recorded spikes.

Importantly, our method provided relatively good estimates of the number of misclassified spikes, estimated by comparing sorted and ground-truth data. Standard solutions to the problem of assessing isolated-cluster quality usually rely on the assumption that spike-shape variations around a waveform template are approximately Gaussian distributed [11]. Although only rarely true for cortical neurons recorded extracellularly [20], it is generally assumed that this simplification holds when spike waveforms are projected into an appropriately chosen feature space. A limitation of this approach is that estimating the posterior probability that a spike belongs to a specific cluster requires estimating the covariance matrix of the clusters along all dimensions of the feature space. When the number of spikes in each cluster is small (~same order as) compared to the number of dimensions, estimating a covariance matrix becomes unreliable. This approach is thus impractical with large multi-electrode arrays when the number of clustered events corresponding to a single potential unit is less than a few hundreds. Techniques exist to deal with this type of problem, mainly by reducing the dimensionality of the space over which the covariance matrix has to be estimated for each cluster [21,22]. Our approach provides another alternative: to use cluster reliability across many runs of the algorithm, so as to obtain an estimate of misclassification probability. This is similar to the stability metrics recently proposed by Barnett et al. [23]. Our method uses this metrics to identify the most consensual clustering solution.

In the datasets we used, a few ground-truth units could not be correctly isolated due to their similarity with spikes from other cells (Fig 7). For some of these, our estimate of the rate of misclassification was off compared with the actual number of misclassified spikes. This is because, like other distance measures (e.g. isolation distance), our metrics rely on the comparison between spikes from different clusters. Single units that are mistakenly merged into the same cluster will therefore appear as well isolated if they are distant enough from the other clusters. Different methods have been proposed to detect such spurious mergers (e.g. standard deviation test, χ^2^ test [10]). However, as this often occurs for spikes that have similar waveforms, it is difficult to distinguish such contaminated clusters from well-isolated single units with variability in their spike shapes.

Another advantage of our method for high-count multi-electrode recordings is that the diversity of spike shapes is mapped onto a lower dimensional space defined by the core clusters, and the distance between clusters is represented as a simple 2-dimensional dendrogram. Visualization of clusters obtained from high-count multi-electrode arrays classically requires selecting a subset of 2 or 3 features (generally defined as principal components of the spike waveform ensemble) to represent the proximity of the identified clusters in a convenient way. But because the optimal feature subset is specific to the clusters that need to be compared, a global inter-cluster distance representation is not possible. By plotting the distance between clusters as a cluster tree, our method allows one to visualize and refine the spike clustering regardless of the dimensionality of the recording. Such dendrograms remain easy to read with up to a few hundred clusters.

Our method typically took 20 minutes to sort ~40,000 spikes obtained from a 32-channel electrode array on a 6-core computer. Because Single-Link clustering runs on the core clusters rather than on single spikes, computation time for hierarchical clustering depended more on the number of units than on the number of spikes. Indeed the diversity of spike shapes matters more than numbers because spikes reliably assigned to the same clusters across all iterations can be processed as single data points. Hence, most of the delay concerned computing the pairwise probabilities that spikes belong to the same clusters across all template-matching iterations (P_0_, see Methods). In practice, the number of groups of spikes that were always assigned to the same cluster across all iterations was still about 80 to 90% of the total number of spikes. For large dataset (containing ≥100,000 spikes), the estimation of the pairwise probability matrix P_0_ might therefore lead to memory issues. A solution could be to apply the method developed here on a subset (*e*.*g*., random) of the spikes and to sort the remainder by template matching, together with the 5% of spikes excluded due to excessive χ^2^ error. Another alternative would be to use as ‘core’ clusters, the clusters obtained for the best clustering solution of all iterations (i.e. with the smallest χ^2^ error). This will reduce the problem to the number of templates K used at each iteration (most likely < 1000, even for large recordings). When applied to ground-truth datasets, this approach considerably reduced memory use and resulted in almost similar performance as when ‘core’ clusters are defined by hierarchical clustering (S7 Fig).

One may consider alternative developments of the algorithm presented here. For instance, the merging of the core clusters by hierarchical clustering could be done with a “greedy approach”, by which the probability matrix of misclassification would be updated after each merging step to take into account the new number of spikes in the newly formed cluster. Although in our hands this approach generally led to an evident over-clustering of the spike waveforms, it might be useful depending on the dataset or the application.

One may also consider that the size threshold on the core clusters (minCsize) should not necessarily be the same for all clusters but instead be adapted to each of them by re-running the consensus clustering phase on each of the identified clusters independently (to find a more ‘optimal’ size threshold).

Finally, it is not uncommon that spike waveforms corresponding to single units drift over the duration of a long recording session, due to slow electrode creep or tissue relaxation. Although our method assumes waveform stationarity in its current form, solutions to the drift problem exist. For example, one could apply consensus clustering only to spikes recorded early and sort the remainder with an adaptive template matching procedure [24]. One could also introduce cluster-shape adaptability over each clustering iteration. In this case, each iteration would consist of an initial clustering of the first spikes and an adaptive template matching for the rest of the recording. In this way, all spikes would be eventually considered for consensus clustering while the cluster labels of each iteration would remain attached to spikes that can drift over time.

In conclusion, while we provide here a comprehensive method for spike sorting (https://github.com/SpikeConsensus), our main contribution resides in that clustering relies simply on an estimate of the misclassification rate from the robustness of the spike clusters and not on any arbitrary statistical model. In principle, this approach could be applied to any other spike sorting technique similar to our template-matching procedure, as long as the convergence of the algorithm depends on some initial conditions. This would allow some standardization across laboratories by providing a common metric (misclassification rate) to assess the quality of the identified single units.

## Methods

The code of our spike sorting toolbox and the data we analyzed are publicly available at https://github.com/SpikeConsensus.

### Datasets

We assessed the accuracy of our spike-sorting method using ground truth datasets, obtained by simultaneous extracellular and intracellular recordings performed either in rat hippocampal CA1 with tetrodes ([9]; hc-1 datasets d14521001, d11221002, d11222001, d12821001, d1122107) or in acute turtle cortical slabs [25] using planar 59 multi-electrode arrays (Multi Channel Systems, Reutlingen, Germany; Mark Shein-Idelson, Lorenz Pammer, Mike Hemberger and Gilles Laurent, unpublished). Ground truth spikes were detected in the intracellular trace by crossing of the voltage-threshold crossing (70% of the action potential peak amplitude).

We also assessed the accuracy of our spike sorting method on artificial 32 channels datasets constructed from the ground truth tetrode recordings. For 4 of the tetrode datasets (d14521001, d11221002, d11222001, d12821001), sustained epochs of stable recordings were selected and replicated 8 times with a time shift (~ 100 ms), resulting in a 32-channel recording with 8 ground-truth spike trains. Those recordings were processed as if they were obtained from a linear 32-electrode array with 20-μm spacing between electrode sites.

Finally, spike sorting was also performed on an *in vivo* recording dataset in which we added to the raw trace artificial spikes at known times. Artificial spikes corresponded to the templates of single units obtained from another recording and whose amplitude was varied randomly by +/- 10% standard deviation to mimic realistic single cell extracellular spike variability. Recordings during visual stimulation were obtained using 32-channel linear silicon probes (20 μm pitch, 172 μm² surface area/site, Neuronexus, Ann Arbor, USA) spanning ~700 μm of the three-layered visual cortex of a lightly anaesthetized (isofluorane, 0.5–1%), paralyzed (pancuronium bromide, 0.2 mg/kg/h) turtle (*Trachemys scripta*,*NASCO Biology*, *Fort Atkinson*, *USA)*. All procedures followed institutional guidelines and were approved by the local authorities (RP Darmstadt, protocol F122/13). The surgical procedure (craniotomy and placement of an intravenous catheter for superfusion) was performed under deep anesthesia (isofluorane, 4–5%) started after induction with Ketamine hydrochloride (CP Pharma, Burgdorf, Germany, 20 mg/kg) and Dexmedethomidine hydrochloride (Dexdomitor, Orion Pharma, Hamburg, Germany, 0.1 mg/kg). At the end of the experiment, the turtle was euthanized by decapitation under deep anaesthesia.

### Detection and preprocessing of spike waveforms

The wide band extracellular signal recorded from each electrode was digitally band-passed filtered between 200 and 4000 Hz. Spikes were first detected independently on each electrode by crossing of the raw signal with a negative threshold defined as [9,26]:

$$T=-z_{th}\times \frac{median(\left|V(t)\right|)}{0.6745}$$ (1)

with z_th_ = 5 (except for d1122107, z_th_ = 6).

Spike times were measured as the time of the trough following threshold crossing. Events separated by times shorter than a censored period (τ_c_, 0.6 ms for hippocampal recordings and 2 ms for turtle cortex recordings) and detected on electrodes less than 60 μm apart (or 3 recording sites) were lumped together, with a spike time corresponding to that of the largest voltage deflection. With this procedure, we defined the spatial window associated with each spike as the smallest contiguous set of electrodes covering all the spike detections that had been lumped together. Spike waveforms were then defined as the band-passed signal recorded across all channels of the array, over a 6-ms time window centered on the spike time. To save storage space and computational time, spike waveforms were resampled around the spike time at the Nyquist frequency of the band-pass filter.

We then performed a spatial whitening of the spike waveforms. From the original recording, we extracted snippets of the same length as the waveforms, but for which no threshold crossing was detected at 4 times the median absolute deviation. Using those snippets, we estimated the spatial covariance matrix of the noise and whitened the spike ensemble by multiplying the waveforms with the square root of the inverse of the covariance matrix [2,27].

Finally, we spatially windowed the whitened spikes by replacing with Gaussian noise, waveforms that were more than 40 μm away (or 2 recording sites) from the edges of the spike spatial window. Note that this spatial windowing left unchanged spike waveforms in tetrode recordings. However, for high-count electrode arrays, we observed that spatial windowing increased the number of significant components recovered during the feature extraction (see next section) and improved overall sorting performances [21].

### Feature extraction

For feature extraction, we considered a temporal window centered on the spike time (τ_win_ = 2.5 ms for the hippocampal recordings and τ_win_ = 4 ms for the turtle recordings). The extracted time-matched spike waveforms were concatenated across channels: each spike was thus represented as a single spatiotemporal vector [10]. We then performed a principal component analysis (PCA) of the spike waveform ensemble and identified the P principal components along which the distribution of projection values of the spike waveforms were not Gaussian (Lilliefors test, p < 0.01) [26]. Each spike was then projected onto these principal components to give a P-dimensional feature vector.

### Consensus-based clustering

The main steps of the proposed consensus clustering procedure are outlined in Table 1.

**Table 1:** Main steps of the consensus-clustering approach for spike sorting.

*1-* Compute $N_{\text{ite}}$ clusterings of the spike waveforms based on the following template matching procedure: - Cluster the spike waveforms in PC space using K-means with K clusters and a new random seed - Compute the average template waveform of each K-means cluster (K-template) - Assign each spike waveform to the best matching K-template according to Eq (2) and measure the corresponding χ^2^. *2-* Select the 95%-tile of the ‘best’ spikes {S_best_} according to their average χ^2^ across all iterations *3-* Compute the probability of pairs of spikes ∈ {S_best_} to be assigned to the same cluster across all iterations (P_0_, Eq 3) *4-* Build the cluster tree over the distance matrix 1 –P_0_ using the Single-link method *5-* Adjust the inconsistency coefficient to cut the tree such as to obtain the smallest possible number of clusters > 1 *→For increasing values of minCsize*, *repeat*: *6-* Reassign spikes from clusters with N_spk_ < minCsize to the closest cluster (‘core’ clusters) with N_spk_ > minCsize, using the distance matrix 1 –P_0_. *7-* Compute the probability of misclassified spikes between pairs of core clusters (P_mis_, Eq 7). *8-* Build the cluster tree over the distance matrix 1 –P_mis_ using the Single-link method. *9-* Cut the cluster tree at 1—P_th_ to obtain the final clusters *→Until the final number of clusters reaches a maximum* *10-* Compute the average template of each *final cluster*. *11-* Assign the remaining 5% of the spikes (that were not in {S_best_}) by recursive template matching to detect putative overlaps.

Spikes waveforms were clustered using a template-matching approach, where templates are identified by K-means clustering of the spikes in feature space.

The feature vectors were clustered into K clusters using a K-means algorithm that minimized, over all clusters, the within-cluster sum of the squared Euclidian distance of spikes to the cluster centroid [5,10]. The template T(x,t) of each K-means cluster was computed as the average of the spatiotemporal waveforms of the spikes belonging to that cluster, over the time window τ_win_. These templates were then fitted to the spike waveforms s(x,t), allowing a ± 20% scaling *a* of the templates amplitude [2]:

$$s_{i}(x,t)=a_{ij}\times T_{j}(x,t)+e_{ij}(x,t)$$ (2)

$$\text{with}\;0.8<a_{ij}<1.2$$

Every spike was eventually assigned to the cluster k* that explained best its spatiotemporal waveform in the least square sense (i.e. k* = *argmin*_*j*_(⟨*e*_*ij*_(*x*,*t*)^2^⟩_*x*,*t*_)). The χ^2^ error in how well the cluster templates explained each spike waveform was defined as ⟨*e*_ik*_(*x*,*t*)^2^⟩_*x*,*t*_.

K-means requires K (the number of clusters) to be set. The procedure described above was applied with K increasingly large and we measured the corresponding χ^2^ errors averaged over all spikes. Larger values of K resulted in lower χ^2^ errors. But as K increased, the improvement in the fit reached a plateau (S3 Fig). As a rule, we chose the smallest value of K < √N_spk_, (where N_spk_ is the number of spike waveforms) to be the closest to this plateau. Nevertheless, the performance of our method appeared stable over a large range of K values (S3 Fig).

The template-matching procedure described above was repeated $N_{\text{ite}}$ times ($N_{\text{ite}}$ = 100, throughout this study), with a new random initial partition for the K-means algorithm at every iteration. Eventually, each spike s_i_ was represented as a vector of integers K_si_, corresponding to the labels of the clusters it was assigned to at successive iterations.

Before proceeding further, we defined a χ^2^ threshold (χ^2^_th_) at 95% of the cumulative distribution of the χ^2^ error values averaged across all $N_{\text{ite}}$ iterations; only spikes with an average χ^2^ error below this threshold were considered for consensus clustering (S2A Fig).

Consensus clustering based on evidence accumulation relies on the assumption that points belonging to true clusters are more likely to be co-localized in the same clusters across an ensemble of $N_{\text{ite}}$ clustering solutions [12,13]. In that framework, the similarity between any two spikes of the dataset can be estimated by measuring their probability P_0_ of being assigned to the same K-means clusters across all $N_{\text{ite}}$ K-means solutions:

$$P_{0}(s_{i},s_{j})=\frac{1}{N_{ite}}\times \sum_{n}Vote(n)$$ (3)

$$where\;Vote(n)=\left\{ \begin{array}{l} 1\;if\;K_{si}(n)=K_{sj}(n)\\ 0\;if\;K_{si}(n)\neq K_{sj}(n) \end{array}\right.$$

Classically, consensus clustering based on evidence accumulation would directly apply a Single-Link method over this probability matrix to merge elements that have a probability higher than a certain threshold to be co-localized in the same K-means clusters. With spike sorting, however, the rate of misclassified spikes is more relevant than the absolute number of misclassifications; the probability of two sets of spikes being merged should therefore take into account the number of spikes contained in each of these subsets. Moreover, because spike data can be noisy and also contain overlapping waveforms, we wanted to avoid small groups of outliers acting as links between distinct single units. We therefore introduced a constraint on the minimum number of spikes (minCsize) that can constitute a single link. Towards this aim, we first performed a hierarchical clustering over the distance matrix defined by 1—P_0_ and adjusted an inconsistency coefficient threshold so as to identify clusters of spikes that were most consistently co-localized in the same K-means cluster. The inconsistency coefficient measures, for each putative link of the cluster tree, the ratio of the height of the link (i.e., the distance between the linked groups of points) to the average height of other links at the same level of the hierarchy [14]. Increasing the inconsistency threshold thus resulted in grouping together more and more spikes. We observed that there always existed a particular value of this coefficient at which all spikes became lumped into a single cluster (S2 Fig). At this threshold, we therefore obtained a partitioning of the spikes based on the consistency of each cluster relative to the rest of the cluster tree. After this first operation, clusters with n_spk_ < minCsize were assigned to the closest cluster with n_spk_ > minCsize provided that they had a probability of being clustered together P_0_ higher than $1/N_{\text{ite}}$ otherwise, spikes were excluded from the consensus clustering procedure. This procedure was repeated with increasing values of the minCsize threshold (typically 3 < minCsize < 20) and we eventually used the parameter value that resulted in the highest number of final clusters (until the number of spikes excluded from the consensus clustering procedure exceeded 0.1% of the total number of spikes). Indeed, we observed that increasing the minCsize threshold usually resulted in increasing the total number of identified final clusters towards a maximal value (S2 Fig). This can be explained as follows: for smaller values of minCsize, more small core clusters can link together distant core clusters; for larger minCsize, groups of spikes which did not pass the size threshold were merged to the closest core cluster with a number of spikes higher than minCsize; therefore the remaining core clusters shared more overlap and tended to be merged together when their overlap reached the misclassification threshold (P_th_). For the data presented here, this procedure resulted in between 50 and 300 clusters depending on the recordings.

By this stage, we had obtained a set of ‘core’ clusters $C_{\text{i}}$, each with a total number of spikes n_spk_ = ${\text{N}}_{Ci}$, greater than minCsize. The probability of misclassifying spikes between any pair of core clusters was then estimated from the number of times that spikes assigned to one were localized in the same K-means clusters as spikes assigned to the other, across all $N_{\text{ite}}$ iterations.

Let *S*_*Ci*_ = {*s* | *s* ∈ *C*_*i*_} be the set of spikes assigned to core cluster $C_{\text{i}}$ and *S*_*k*_(*n*) = {*s* | *K*_*s*_(*n*) = *k*}, the set of spikes assigned to cluster k at the n^th^ iteration. For every $N_{\text{ite}}$ iteration n, we define the ensemble of misclassified spikes between core clusters $C_{\text{i}}$ and $C_{\text{j}}$ as:

$${mis}_{n}(C_{i},C_{j})=\underset{k}{⋃}\left({FP}_{n}^{C_{i},C_{j}}(k)\cup {FN}_{n}^{C_{i},C_{j}}(k)\right)$$ (4)

where

$${FP}_{n}^{C_{i},C_{j}}(k)=S_{Ci}\cap S_{k}(n)\;\text{if}\;|S_{Ci}\cap S_{k}|<|S_{Cj}\cap S_{k}|$$ (5)

and

$${FN}_{n}^{C_{i},C_{j}}(k)=S_{Cj}\cap S_{k}(n)\;\text{if}\;|S_{Cj}\cap S_{k}|<|S_{Ci}\cap S_{k}|$$ (6)

(The |…| operator denotes the number of elements in the ensemble.) In other words, we considered as potential false positive (alternatively, negative) classifications, cases in which spikes assigned to $C_{\text{i}}$ (alternatively, $C_{\text{j}}$) had been assigned to the same cluster as spikes assigned to $C_{\text{j}}$ (alternatively, $C_{\text{i}}$) at the k^th^ iteration. The condition on the relative sizes of the intersection between core and K-means clusters (|*S*_*Ci*_ ∩ *S*_*k*_| vs. |*S*_*Cj*_ ∩ *S*_*k*_|) reflects the fact that we identified cluster k as representing most commonly either $C_{\text{i}}$ or $C_{\text{j}}$, depending on the overlap with either of them. Note that because ${FP}_{n}^{C_{i},C_{j}}(k)$ = ${FN}_{n}^{C_{j},C_{i}}(k)$, *mis*_*n*_(*C*_*i*_,*C*_*j*_) = *mis*_*n*_(*C*_*j*_,*C*_*i*_).

The probability of misclassification between two core clusters was then defined as

$$P_{mis}(Ci,Cj)=\frac{1}{{\text{N}}_{Ci}+{\text{N}}_{Cj}}\times \frac{1}{N_{ite}}\times \sum_{n=1}^{N_{ite}}\left|{mis}_{n}(C_{i},C_{j})\right|$$ (7)

A Single Link method was applied over the distance matrix defined by 1 –P_mis_ and the resulting dendrogram was cut at a threshold 1 –P_th_, thereby merging into the same ‘final’ cluster U, neighboring core clusters C that had pairwise probabilities of misclassified spikes higher than P_th_. Once the final clusters had been obtained, we updated the pairwise misclassification probability with the new number of spikes contained in each final cluster:

$$P_{mis}(Ui,Uj)=\frac{1}{{\text{N}}_{Ui}+{\text{N}}_{Uj}}\times \frac{1}{N_{ite}}\times \sum_{n=1}^{N_{ite}}\left|{mis}_{n}(U_{i},U_{j})\right|$$ (8)

We then assigned the remaining 5% of the spikes initially excluded from the consensus clustering procedure due to their excessive χ^2^ average, using a “greedy” matching pursuit approach similar to strategies proposed by others before [2,28,29]. For each one of these spikes, we identified the cluster $U_{1}*$ that best explained its spatiotemporal waveform according to Eq (2). If the corresponding χ^2^ residual was below the previously defined χ^2^ threshold, the spike was assigned to $U_{1}*$. If the corresponding χ^2^ residual exceeded the previously defined χ^2^ threshold, we considered the spike as a possible overlap and looked for the best overlapping cluster and the best temporal delay, by fitting the cluster templates to the residual ${\text{e}}_{U_{1}*}$ for all possible delays τ:

$$e_{U_{1}*}(x,t)=a_{Ui}\times T_{Ui}(x,t-\tau )+e_{Ui}(x,t)$$ (9)

$$\text{with}\;0.8<a_{ij}<1.2\;\text{and}\;\frac{-\tau_{wf}}{2}<\tau <\frac{\tau_{wf}}{2}$$

If the χ^2^ of the smallest residual still exceeded threshold, we repeated the procedure on the residual of the best overlapping template. If the latter residual was below threshold, the spike was finally validated as an overlap of spikes and assigned to the identified overlapping clusters, provided that their corresponding time shift (τ) was smaller than the censored period (τ_c_) (in which case they had already been detected). If after three iterations, the residual still exceeded the χ^2^ threshold, we considered the spike as non-classified.

### Validation and quality measurements

Not all single units have the same variability in their spike shape: some neurons generate spikes with higher amplitude fluctuation than others, and bursting neurons generate spikes whose shape depends on their rank in the train. For this reason, our procedure could still keep apart clusters of spikes corresponding to the same single unit. To detect these spurious cuts, we applied classical criteria to detect bursting single units [9,29] and flagged pairs of clusters that had a normalized cross-correlation coefficient above 0.70 and a pairwise probability of misclassified spikes (P_mis_) higher than 0.05 for visual inspection.

To quantify the overall quality of the identified single units Ui, we estimated the total probability of false positive (FP) and false negative (FN) misclassification, by measuring the number of times spikes in Ui ($S_{Ui}=\{s|s\in u_{i}\}$) were co-clustered with other spikes ($S_{∼Ui}=\{s|s\notin u_{i}\}$) across all $N_{\text{ite}}$ clustering iterations:

$$P_{FP}^{total}(Ui)=\frac{1}{{\text{N}}_{Ui}}\times \frac{1}{N_{ite}}\times \sum_{n=1}^{N_{ite}}\left|\underset{k}{⋃}\left({FP}_{n}^{Ui}(k)\right)\right|$$ (10)

$$P_{FN}^{total}(Ui)=\frac{1}{{\text{N}}_{Ui}}\times \frac{1}{N_{ite}}\times \sum_{n=1}^{N_{ite}}\left|\underset{k}{⋃}\left({FN}_{n}^{Ui}(k)\right)\right|$$ (11)

where

$${FP}_{n}^{Ui}(k)=S_{Ui}\cap S_{k}(n)\;\text{if}\;|S_{Ui}\cap S_{k}|<|S_{∼Ui}\cap S_{k}|$$ (12)

and

$${FN}_{n}^{Ui}(k)=S_{∼Ui}\cap S_{k}(n)\;\text{if}\;|S_{∼Ui}\cap S_{k}|<|S_{Ui}\cap S_{k}|$$ (13)

Single units were validated when they had a total rate of misclassified spikes ($P_{FP}^{total}+P_{FN}^{total}$) lower than 20%.

The signal-to-noise ratio (SNR) of each single unit was measured as the maximal SNR measured from the single-unit template across space and time:

$$SNR(Ui)={max}_{x,t}\left(\sqrt{\frac{{T_{Si}(x,t)}^{2}}{{Var}_{Si}(S_{Ui}(x,t))}}\right)$$ (14)

Our consensus clustering algorithm tended to lump together small amplitude spike waveforms. Although these clusters corresponded to multi-unit activity, their corresponding total rate of misclassification ($P_{FP}^{total}+P_{FN}^{total}$) could fall below 20%, because no other single unit was so small as to contaminate them. Therefore, we considered identified clusters as putative single units only when their SNR was higher than 4.

For comparison purpose, we also measured the isolation distance for the tetrode recordings [9], which we measured in a feature space defined by the 12 first principal components of the concatenated spike waveforms (see feature extraction section).

### Validation with “Ground-Truth” Datasets

To estimate the performances of the spike sorting on the ground truth datasets, we considered that a detected extracellular spike matched a ground truth spike when it occurred within a 2 ms window around the time of an intracellular action potential. Ground truth spikes that could not be assigned to any extracellular spike were counted as false negative detection (FN_d_). For each dataset, we identified the single unit with the highest count of matches with the ground truth and calculated the true rate of false positives and false negatives relative to the number of ground truth spikes detected in the extracellular trace:

$${FP}_{rate}=\frac{FP}{FN-{FN}_{d}+TP}$$ (15)

$${FN}_{rate}=\frac{FN-{FN}_{d}}{FN-{FN}_{d}+TP}$$ (16)

where

FP = number of false positive spikes, i.e. spike times with no match in the ground truth

FN = number of false negative spikes, i.e. ground truth with no match in the spike times

TP = number of true positive spikes, i.e. spikes times with match in ground truth spikes

FN_d_ = number of ground truth spikes not detected in the extracellular trace

To facilitate the comparison with the percentage of misclassified spikes estimated by consensus clustering, we also defined the percentage of false positive and false negative as:

$$\%\;FP=\frac{FP}{FP+TP}$$ (17)

$$\%\;FN=\frac{FN}{FP+TP}$$ (18)

### Support vector machine performances

The performance of our spike sorting method was compared with the best performance obtained with a quadratic support vector machine (SVM) trained on the ground truth data, using a 20-fold cross-validation procedure (Matlab, Statistics Toolbox). Practically, we used the same whitened spike waveform ensemble as the one used in our spike sorting procedure. The SVM was trained and validated over a large range of parameters, exploring different combinations of C-margin, kernel scale and misclassification costs values [21]. The optimal performances of the SVM classifier were measured as in Eqs 15 and 16, for the combination of SVM parameters leading to the smallest total number of misclassified spikes (min(FN_SVM_ rate + FP_SVM_ rate)).

## Supporting Information

S1 Fig K-means clustering and Single-link clustering. a. Partition obtained through K-means clustering when the number of clusters to identify is set to 5. b. Position of the boundaries of the K-means clusters averaged across 200 hundred iterations of K-means, with a number of cluster set to √N, with N = number of data points. The size and color of the symbols scale with the probability that each point falls on the convex hull of a K-means cluster across all iterations. c. Dendrogram obtained by hierarchical clustering with the Single-link method over the matrix defined as the pairwise distance between points, measured in the original dimensions of the dataset. d. Partition obtained by cutting the dendrogram in c at 0.95 (horiz. purple line in c). The five clusters do not match the initial partition of the data: 3 of the clusters are merged together due to the Single-link effect. e. The five initial clusters of the dataset are distinguishable (among 51 other small clusters) when the dendrogram is cut at 0.5 (pink line in c). Consensus-based clustering has the advantage over direct hierarchical clustering that the distance between points corresponds to probabilities of misclassification across many K-means clustering solutions. This metrics is therefore more easily interpretable than true distance in feature space and facilitates the selection of a specific distance at which the cluster tree must be cut.
(TIF)

S2 Fig Successive stages of the consensus clustering procedure. a. Cumulative distribution of χ^2^ error values averaged across all clustering iterations. Only spikes with an average error lower than the χ^2^ value at 95% of the cumulative distribution were considered for consensus clustering. The remaining 5% of the spikes were fitted separately using the final cluster templates identified by consensus clustering (see Methods). b. Cluster tree obtained by hierarchical clustering over the distance matrix defined by 1 –P_0_ (see Methods). The leaves of the tree correspond to groups of spikes that were systematically clustered in the same K-means clusters across all iterations. Out of 6420 spikes used for consensus clustering, we found 4915 such groups of spikes that were systematically clustered together. c. Many of the groups of co-clustered spikes comprised just one or two spikes. Before cutting the cluster tree at a certain height, we adjusted an inconsistency threshold in order to cluster together spikes that were more consistently clustered together than their neighbors. The inconsistency coefficient measures, for each putative link of the cluster tree, the ratio of the height of the link (i.e., the distance between the linked groups of points) to the average height of other links at the same level of the hierarchy [14]. There always existed a particular value of this coefficient (red arrow) above which all spikes became lumped into a single cluster. After this procedure, we obtained a set of ‘core’ clusters (3320 in this example), corresponding to spikes that were most consistently clustered together relative to the rest of the spike ensemble. d. Distribution of core cluster sizes. After applying the inconsistency threshold, more than 2000 core clusters (2150) were still singletons. e. Number of final clusters identified by consensus clustering depending on the core cluster size threshold (minCsize, see Methods). We used the size threshold leading to the highest number of final clusters. f. Distribution of the size of the final clusters, identified by cutting the cluster tree of the core cluster (d) at 1-P_th_, with P_th_ = 0.15.
(TIF)

S3 FigParameters of consensus clustering and sorting performance.a. The χ^2^ error averaged over all spikes (dataset as in Fig 2) for one single iteration of our K-means based template-matching procedure (*black*, *left scale*) decreases as a function of the number of clusters K. This improvement in fit quality (measured by the derivative of the mean χ^2^ error (Δχ^2^, *grey*, *right scale*)) reached a plateau as K increased and we chose the value of K < √Nspk that was the closest to this plateau. b.Sorting performance of our consensus clustering method for different values of Pmis (*top*) or $N_{\text{ite}}$ (*bottom*) and K (same dataset as in Fig 2). Although we proposed a method to guide the choice of K, performance was stable over a large range of value of K as long as Pmis and $N_{\text{ite}}$ were large enough. c. Number of identified clusters that contained ground truth spikes for different values of Pmis (*top*) or $N_{\text{ite}}$ (*bottom*) and K (same dataset as in Fig 2).The number of clusters increased for higher values of K and Pmis, therefore requiring more manual intervention. d,e,f. same as a,b,c for another tetrode dataset where the groundtruth spikes had small amplitudes, close to multiunit activity.(TIF)

S4 Fig Gaussian mixture clustering. a. The spike waveforms of the tetrode recording shown in Fig 2 were clustered in the same N-dimensional feature space as used for our consensus clustering procedure but using an Expectation–Maximization (EM) algorithm to optimize a N-dimensional Gaussian mixture model. The optimal number of Gaussians was defined by selecting the clustering that gave the minimal value (n = 10) for the Bayesian Information criterion. b. This approach failed to identify correctly a substantial number of ground-truth spikes: although one cluster (*left*) matched 87% (157 / 181) of the intracellularly detected spikes with no contamination from other units (% FP = 0), 13% (24 / 181) of the ground-truth spikes could not be separated from other spikes clustered into a large multi-unit cluster (*right*). c. Using the same number of Gaussians in the Gaussian mixture model as the number of clusters identified by consensus clustering (n = 26) did not result in a better clustering of the ground truth spikes: although 84% (152 / 181) of the ground-truth spikes were correctly isolated without contamination (*left*), 16% (29 / 181) of them were still misclassified with other spikes in a non-matching cluster (*right*).
(TIF)

S5 Fig Comparison of consensus clustering performance to optimized K-means template matching. a. Spike sorting performance were quite similar between the consensus clustering method we proposed (consensus-based clustering) and a single iteration of template matching based on K-means (optimized K-means), with the K-means algorithm optimized to find the global rather than local maximum. The optimized K-means used 100 replicates and ‘online phase updates’, where each data point is assessed and re-assigned such as the re-assignment decreases the total sum of distance (Matlab). b. The optimized K-means method however resulted in many more splits of the ground truth spikes in separate clusters than our consensus clustering method, therefore requiring more manual intervention. Contrary to our consensus-based approach, this manual intervention could not be guided readily by any metrics of the actual distance between clusters to be merged.
(TIF)

S6 Fig Comparison of the estimated percentage of misclassified spikes to the Isolation distance. The Isolation distance was measured for the clusters identified in all tetrode recordings, in the feature space defined by the first 12 principal components of the concatenated spike waveforms (see Methods). The comparison between the isolation distance and the percentage of misclassified spike estimated by consensus clustering shows that the latter is more sensitive than the former: as the rate of misclassified spikes increases, the isolation distance drops faster than the estimated percentage of misclassification.
(TIF)

S7 Fig Sorting performance and method to define the ‘core’ clusters. Sorting performances were similar when core clusters were defined by adjusting the inconsistency coefficient over the distance matrix defined by 1—P_0_ (inconsistency coefficient, see Methods) as when we instead used the clusters obtained for the best clustering iteration (i.e. with the smallest average χ^2^ error, best clustering iteration).
(TIF)
