# Supplementary figures and images for: Consensus-Based Sorting of Neuronal Spike Waveforms
Source: PLoS One. 2016 Aug 18;11(8):e0160494. doi: 10.1371/journal.pone.0160494 (PMC4990262; doi:10.1371/journal.pone.0160494)

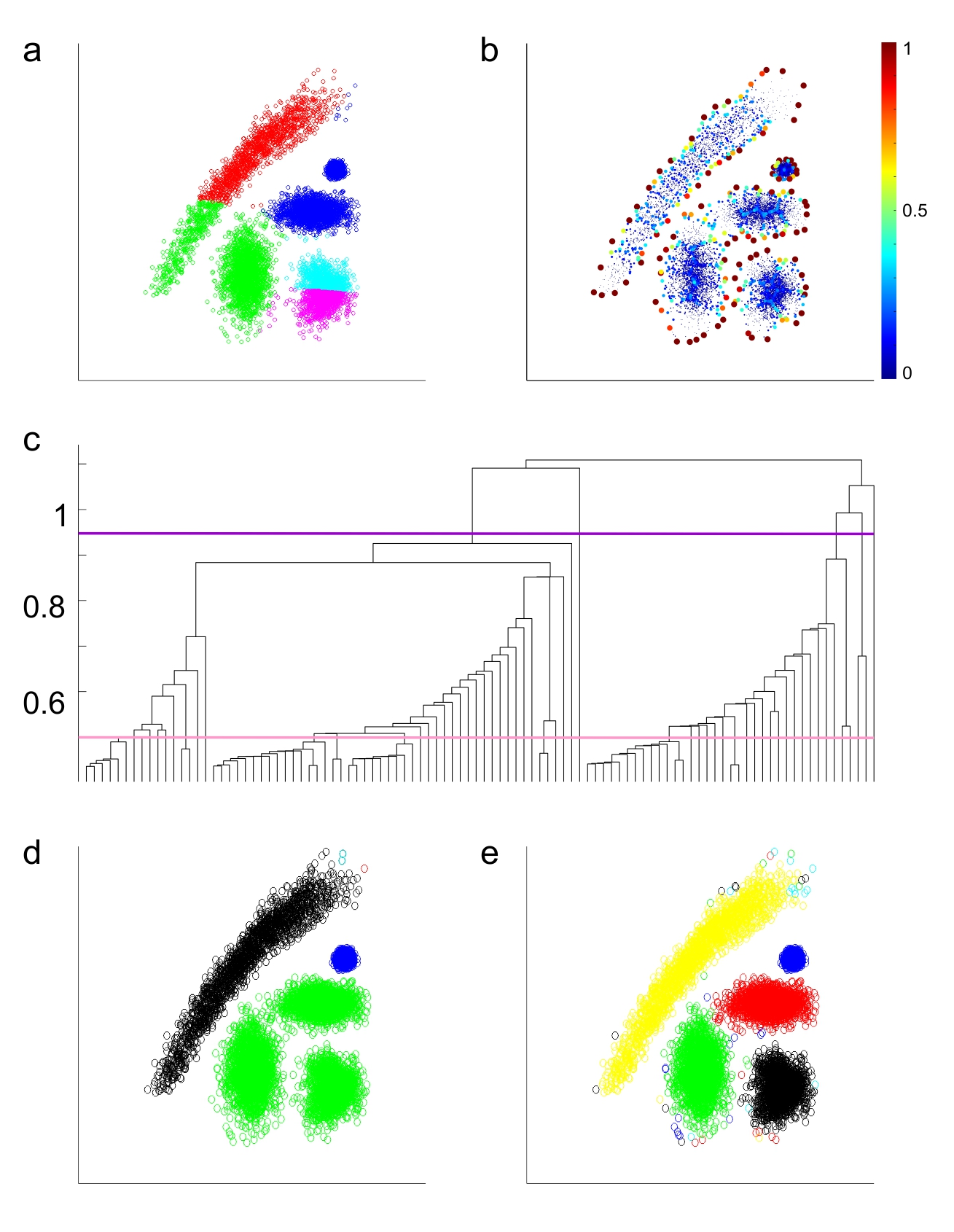

Supplement: S1 Fig — a. Partition obtained through K-means clustering when the number of clusters to identify is set to 5. b. Position of the boundaries of the K-means clusters averaged across 200 hundred iterations of K-means, with a number of cluster set to √N, with N = number of data points. The size and color of the symbols scale with the probability that each point falls on the convex hull of a K-means cluster across all iterations. c. Dendrogram obtained by hierarchical clustering with the Single-link method over the matrix defined as the pairwise distance between points, measured in the original dimensions of the dataset. d. Partition obtained by cutting the dendrogram in c at 0.95 (horiz. purple line in c). The five clusters do not match the initial partition of the data: 3 of the clusters are merged together due to the Single-link effect. e. The five initial clusters of the dataset are distinguishable (among 51 other small clusters) when the dendrogram is cut at 0.5 (pink line in c). Consensus-based clustering has the advantage over direct hierarchical clustering that the distance between points corresponds to probabilities of misclassification across many K-means clustering solutions. This metrics is therefore more easily interpretable than true distance in feature space and facilitates the selection of a specific distance at which the cluster tree must be cut. (TIF) [file pone.0160494.s001.tif]

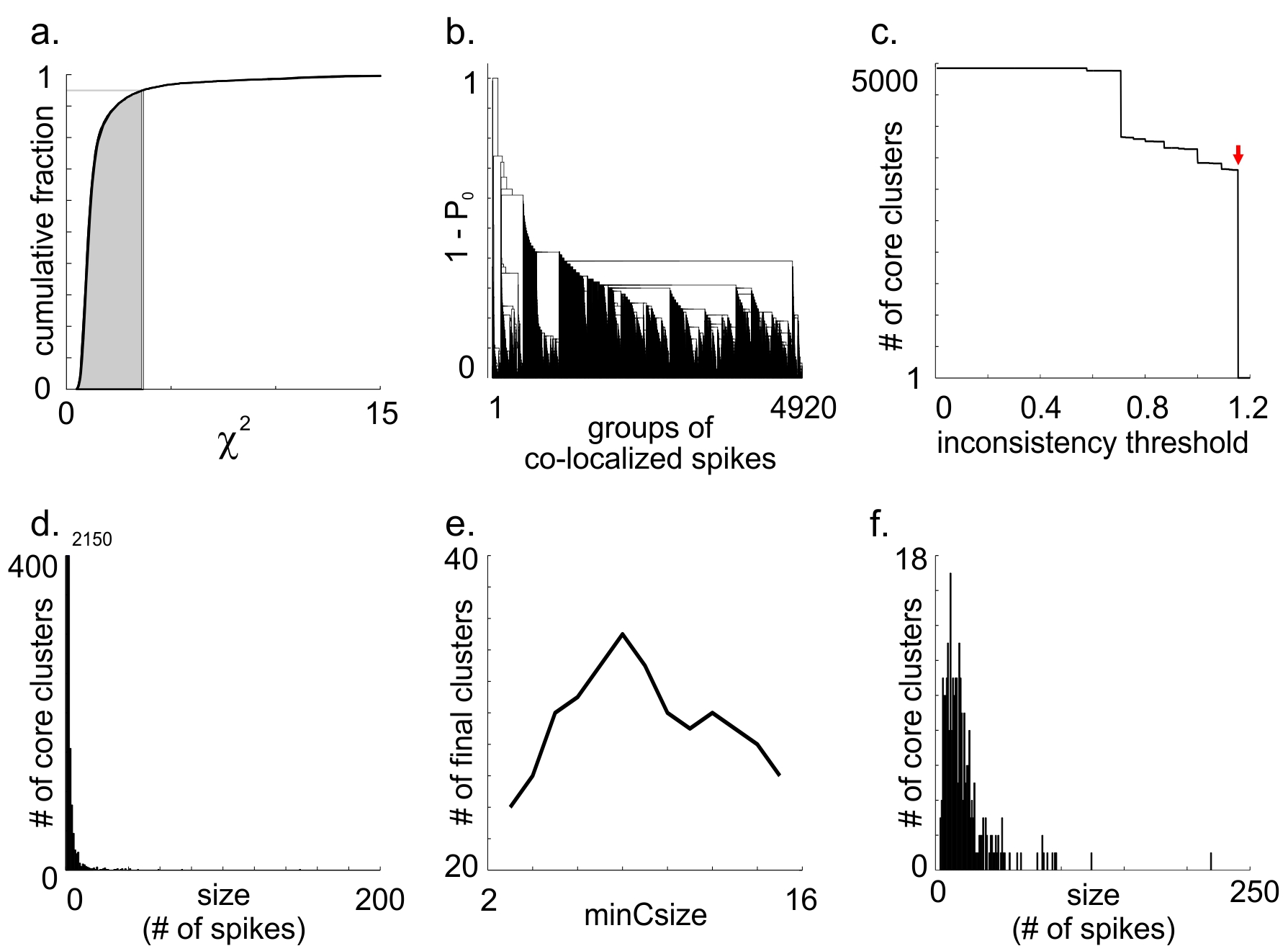

Supplement: S2 Fig — a. Cumulative distribution of χ2 error values averaged across all clustering iterations. Only spikes with an average error lower than the χ2 value at 95% of the cumulative distribution were considered for consensus clustering. The remaining 5% of the spikes were fitted separately using the final cluster templates identified by consensus clustering (see Methods). b. Cluster tree obtained by hierarchical clustering over the distance matrix defined by 1 –P0 (see Methods). The leaves of the tree correspond to groups of spikes that were systematically clustered in the same K-means clusters across all iterations. Out of 6420 spikes used for consensus clustering, we found 4915 such groups of spikes that were systematically clustered together. c. Many of the groups of co-clustered spikes comprised just one or two spikes. Before cutting the cluster tree at a certain height, we adjusted an inconsistency threshold in order to cluster together spikes that were more consistently clustered together than their neighbors. The inconsistency coefficient measures, for each putative link of the cluster tree, the ratio of the height of the link (i.e., the distance between the linked groups of points) to the average height of other links at the same level of the hierarchy [14]. There always existed a particular value of this coefficient (red arrow) above which all spikes became lumped into a single cluster. After this procedure, we obtained a set of ‘core’ clusters (3320 in this example), corresponding to spikes that were most consistently clustered together relative to the rest of the spike ensemble. d. Distribution of core cluster sizes. After applying the inconsistency threshold, more than 2000 core clusters (2150) were still singletons. e. Number of final clusters identified by consensus clustering depending on the core cluster size threshold (minCsize, see Methods). We used the size threshold leading to the highest number of final clusters. f. Distribution of the size of the final c [file pone.0160494.s002.tif]

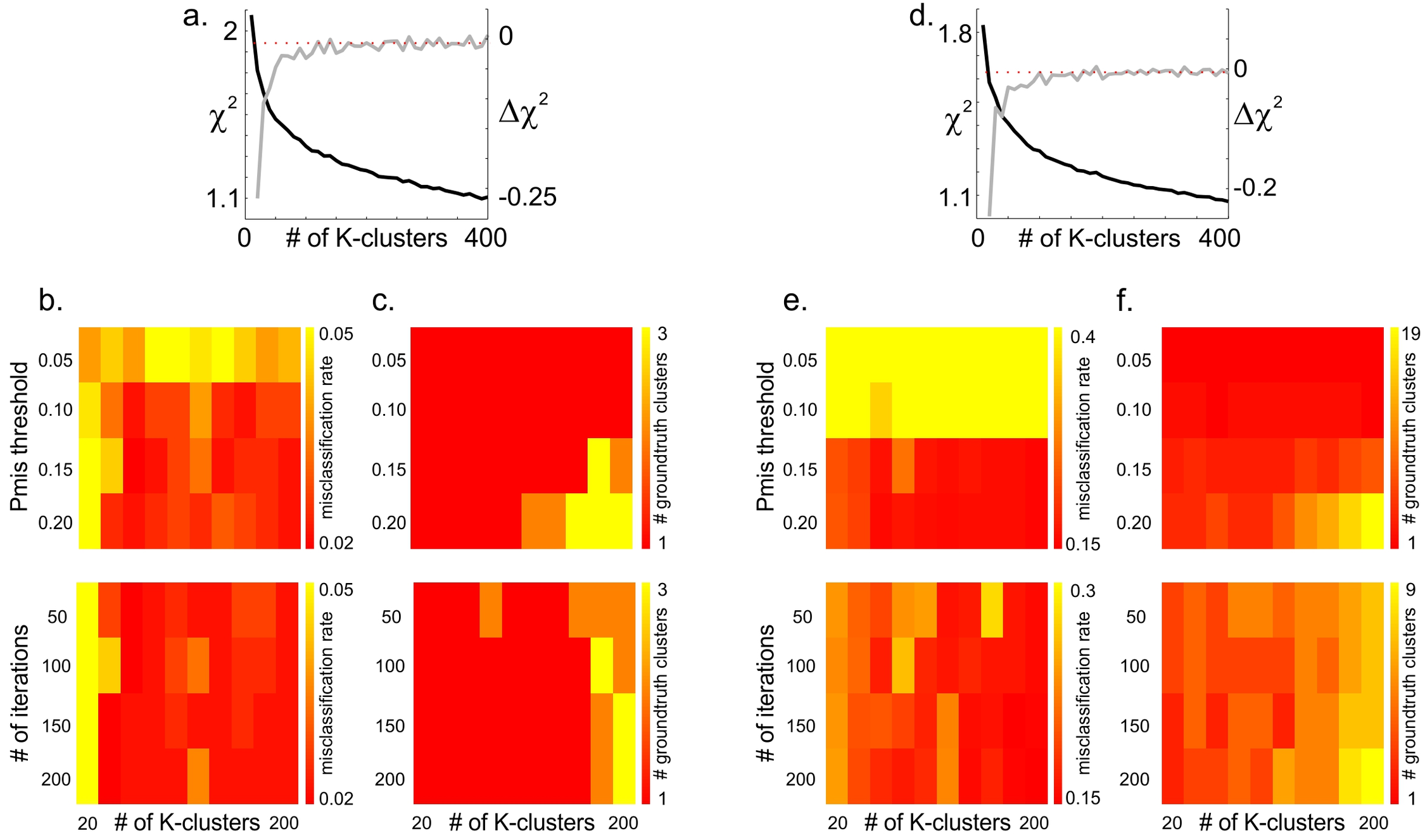

Supplement: S3 Fig — a. The χ2 error averaged over all spikes (dataset as in Fig 2) for one single iteration of our K-means based template-matching procedure (black, left scale) decreases as a function of the number of clusters K. This improvement in fit quality (measured by the derivative of the mean χ2 error (Δχ2, grey, right scale)) reached a plateau as K increased and we chose the value of K < √Nspk that was the closest to this plateau. b.Sorting performance of our consensus clustering method for different values of Pmis (top) or Nite (bottom) and K (same dataset as in Fig 2). Although we proposed a method to guide the choice of K, performance was stable over a large range of value of K as long as Pmis and Nite were large enough. c. Number of identified clusters that contained ground truth spikes for different values of Pmis (top) or Nite (bottom) and K (same dataset as in Fig 2).The number of clusters increased for higher values of K and Pmis, therefore requiring more manual intervention. d,e,f. same as a,b,c for another tetrode dataset where the groundtruth spikes had small amplitudes, close to multiunit activity. (TIF) [file pone.0160494.s003.tif]

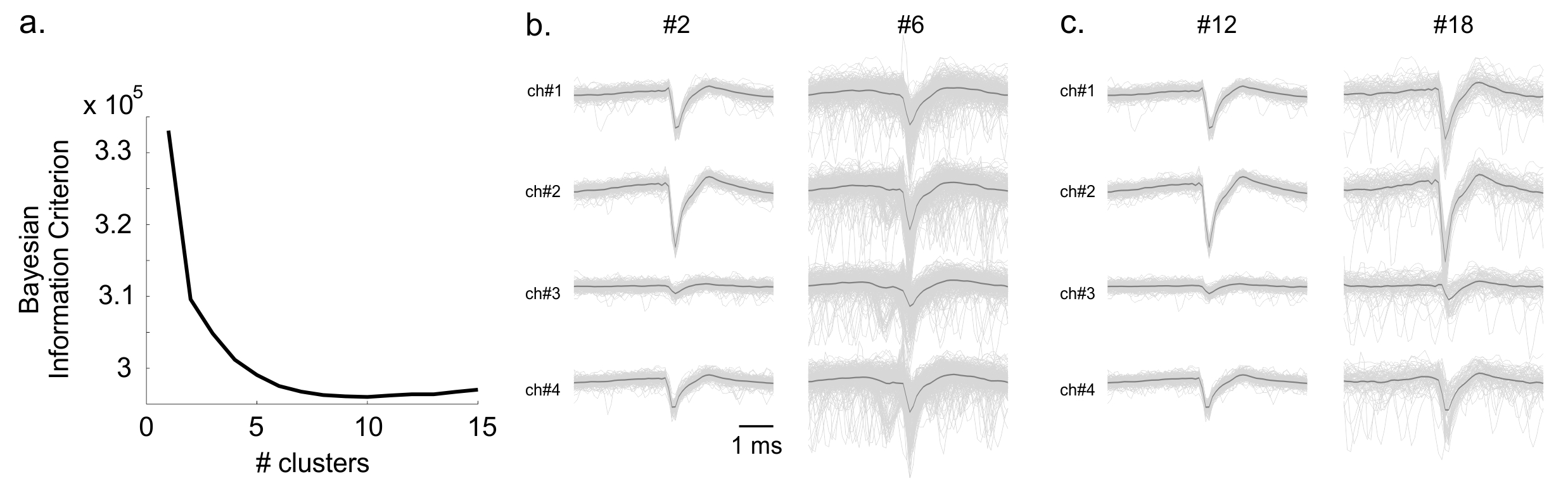

Supplement: S4 Fig — a. The spike waveforms of the tetrode recording shown in Fig 2 were clustered in the same N-dimensional feature space as used for our consensus clustering procedure but using an Expectation–Maximization (EM) algorithm to optimize a N-dimensional Gaussian mixture model. The optimal number of Gaussians was defined by selecting the clustering that gave the minimal value (n = 10) for the Bayesian Information criterion. b. This approach failed to identify correctly a substantial number of ground-truth spikes: although one cluster (left) matched 87% (157 / 181) of the intracellularly detected spikes with no contamination from other units (% FP = 0), 13% (24 / 181) of the ground-truth spikes could not be separated from other spikes clustered into a large multi-unit cluster (right). c. Using the same number of Gaussians in the Gaussian mixture model as the number of clusters identified by consensus clustering (n = 26) did not result in a better clustering of the ground truth spikes: although 84% (152 / 181) of the ground-truth spikes were correctly isolated without contamination (left), 16% (29 / 181) of them were still misclassified with other spikes in a non-matching cluster (right). (TIF) [file pone.0160494.s004.tif]

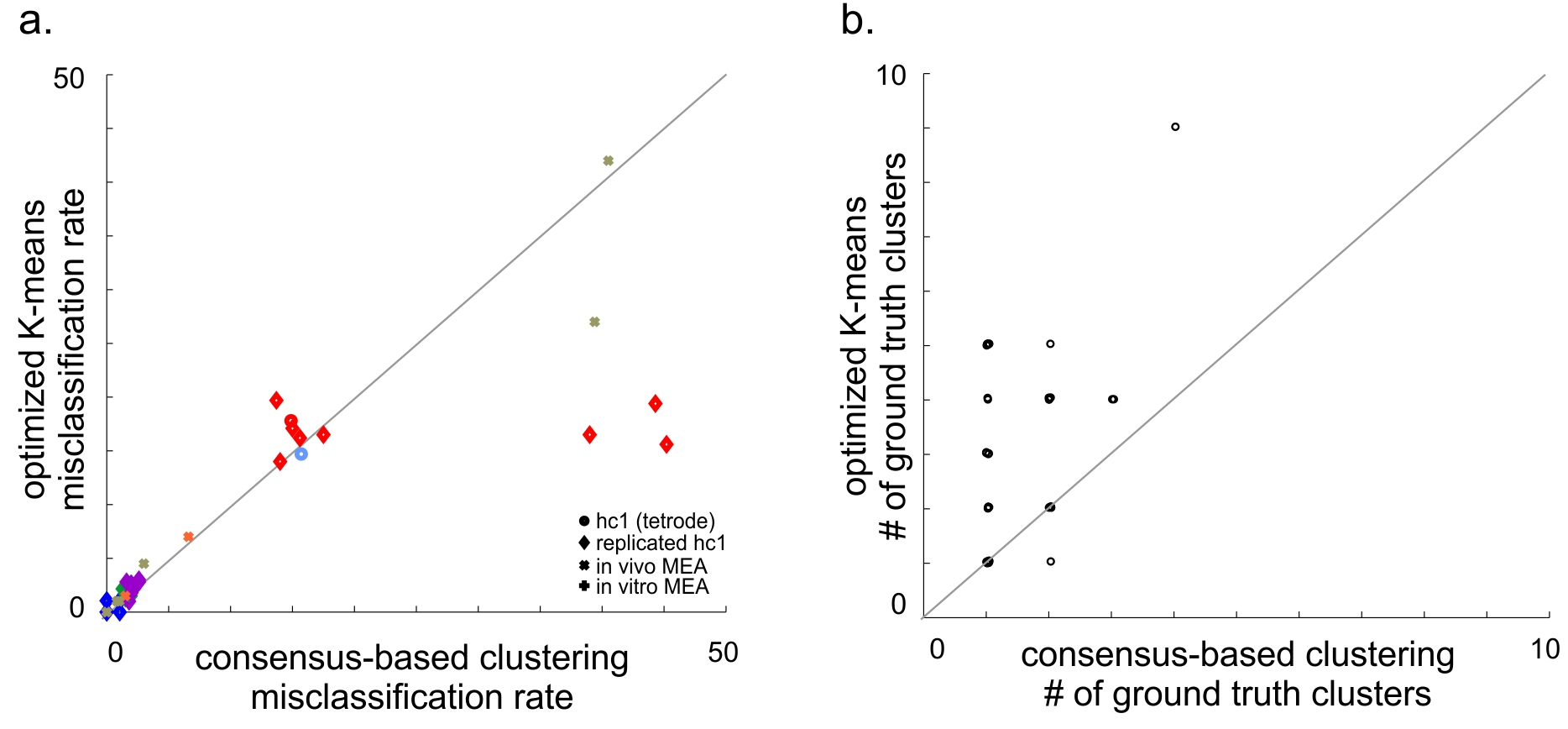

Supplement: S5 Fig — a. Spike sorting performance were quite similar between the consensus clustering method we proposed (consensus-based clustering) and a single iteration of template matching based on K-means (optimized K-means), with the K-means algorithm optimized to find the global rather than local maximum. The optimized K-means used 100 replicates and ‘online phase updates’, where each data point is assessed and re-assigned such as the re-assignment decreases the total sum of distance (Matlab). b. The optimized K-means method however resulted in many more splits of the ground truth spikes in separate clusters than our consensus clustering method, therefore requiring more manual intervention. Contrary to our consensus-based approach, this manual intervention could not be guided readily by any metrics of the actual distance between clusters to be merged. (TIF) [file pone.0160494.s005.tif]

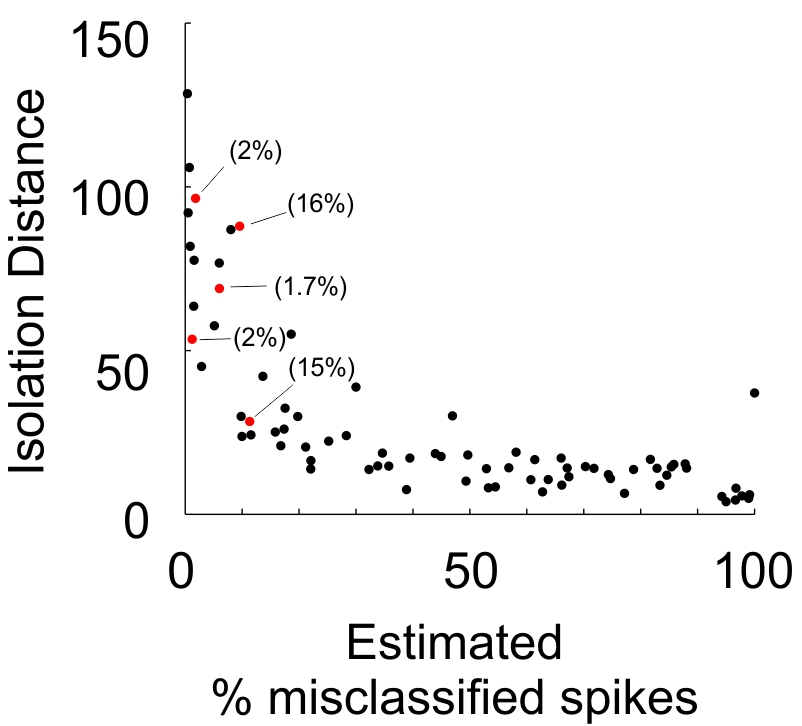

Supplement: S6 Fig — The Isolation distance was measured for the clusters identified in all tetrode recordings, in the feature space defined by the first 12 principal components of the concatenated spike waveforms (see Methods). The comparison between the isolation distance and the percentage of misclassified spike estimated by consensus clustering shows that the latter is more sensitive than the former: as the rate of misclassified spikes increases, the isolation distance drops faster than the estimated percentage of misclassification. (TIF) [file pone.0160494.s006.tif]

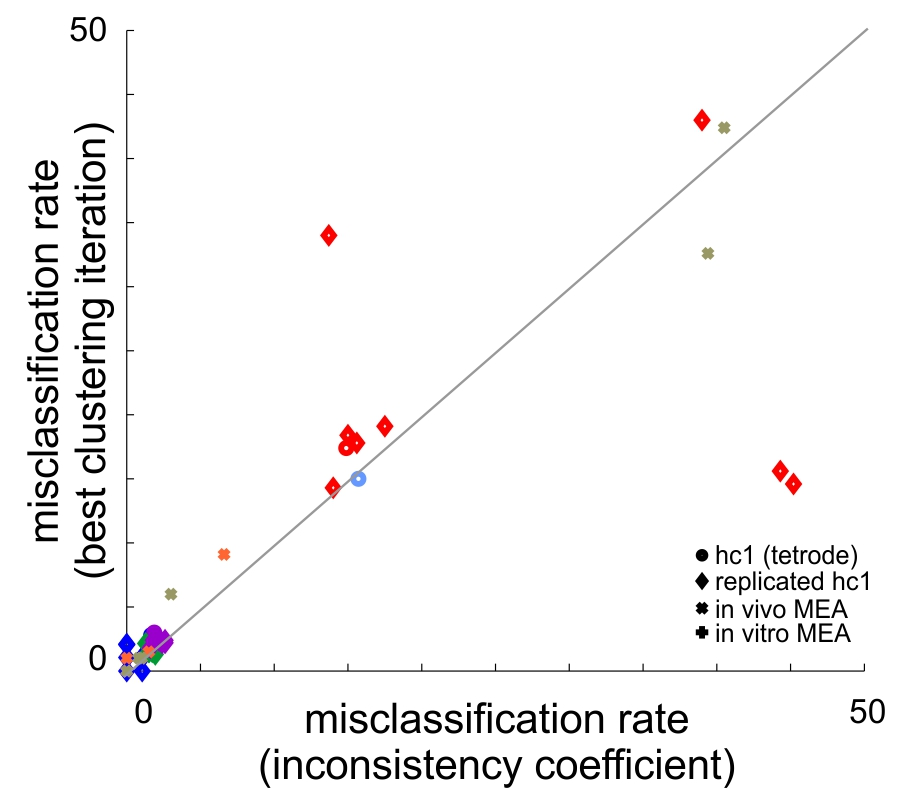

Supplement: S7 Fig — Sorting performances were similar when core clusters were defined by adjusting the inconsistency coefficient over the distance matrix defined by 1—P0 (inconsistency coefficient, see Methods) as when we instead used the clusters obtained for the best clustering iteration (i.e. with the smallest average χ2 error, best clustering iteration). (TIF) [file pone.0160494.s007.tif]
